# Conserving herbivorous and predatory insects in urban green spaces

**DOI:** 10.1038/srep40970

**Published:** 2017-01-19

**Authors:** Luis Mata, Caragh G. Threlfall, Nicholas S. G. Williams, Amy K. Hahs, Mallik Malipatil, Nigel E. Stork, Stephen J. Livesley

**Affiliations:** 1 Interdisciplinary Conservation Science Research Group, School of Global, Urban and Social Studies, RMIT University, Melbourne 3000, Victoria, Australia; 2 School of Ecosystem and Forest Sciences, Faculty of Science, The University of Melbourne, Richmond 3121, Victoria, Australia; 3 Australian Research Centre for Urban Ecology, Royal Botanic Gardens Victoria c/o School of BioSciences, The University of Melbourne, Parkville 3010, Victoria, Australia; 4 Department of Economic Development, Jobs, Transport and Resources, AgriBio, La Trobe University, Bundoora 3083, Victoria, Australia; 5 Environmental Futures Research Institute, Griffith School of Environment, Griffith University, Nathan 4111, Queensland, Australia

## Abstract

Insects are key components of urban ecological networks and are greatly impacted by anthropogenic activities. Yet, few studies have examined how insect functional groups respond to changes to urban vegetation associated with different management actions. We investigated the response of herbivorous and predatory heteropteran bugs to differences in vegetation structure and diversity in golf courses, gardens and parks. We assessed how the species richness of these groups varied amongst green space types, and the effect of vegetation volume and plant diversity on trophic- and species-specific occupancy. We found that golf courses sustain higher species richness of herbivores and predators than parks and gardens. At the trophic- and species-specific levels, herbivores and predators show strong positive responses to vegetation volume. The effect of plant diversity, however, is distinctly species-specific, with species showing both positive and negative responses. Our findings further suggest that high occupancy of bugs is obtained in green spaces with specific combinations of vegetation structure and diversity. The challenge for managers is to boost green space conservation value through actions promoting synergistic combinations of vegetation structure and diversity. Tackling this conservation challenge could provide enormous benefits for other elements of urban ecological networks and people that live in cities.

Urbanisation has caused, and is forecasted to increasingly cause, global detrimental impacts on biodiversity^1^,^2^. Yet, a mounting body of evidence suggests that urban environments can still support substantial levels of native biodiversity including many threatened species^3^. Cities therefore provide unique opportunities to proactively implement actions and strategies to conserve biodiversity. These actions may also have significant benefits for people, as biodiverse urban ecosystems are known to improve the health and wellbeing of city-dwellers^4^. However, successful conservation and management strategies relevant to a range of functionally different taxa are yet to be devised, and much guidance is still required for conservation practice to realise the opportunities that biodiverse urban areas could provide.

Insects are a key component of urban biodiversity^5^, and the ecological functions they perform translate into a wide array of ecosystem services^6^,^7^ as well as disservices^8^,^9^. In forest ecosystems, Ewers *et al*.^10^ showed that the contribution of insect and other invertebrate taxa to litter decomposition, seed predation and invertebrate predation was halved following logging because of a significant decrease in invertebrate abundance. Furthermore, insects play a key ecological role as prey for other taxa - especially insectivorous birds, reptiles and microbats. In urban ecosystems, it is poorly understood how insect diversity is affected by changes to vegetation during and after urbanisation. Investigating how key functional groups, such as herbivores and predators^11^, respond to changes in vegetation structure and diversity may help develop this understanding^12^ and help determine what strategies may best conserve these functional groups and the ecosystem services they provide.

Heteropteran bugs (Hemiptera: Heteroptera; henceforth bugs for brevity) comprise a hyperdiverse monophyletic clade of insects distributed worldwide^13^,^14^. Bugs present a wide range of feeding strategies from strict phytophagy and zoophagy to omnivory^15^,^16^, making them a suitable model taxon to better understand responses of insect herbivores and predators to gradients in urban vegetation structure and diversity. Bugs also provide important ecosystem services. For example, generalist predators such as damsel, pirate and assassin bugs (families: Nabidae, Anthocoridae and Reduviidae) are biological control agents in forest and agricultural ecosystems^15^,^17^. Finally, some herbivorous bug species are highly host plant specific. For example, 60% of mirid bugs (family Miridae) are associated exclusively with a single host plant and less than 20% occur in more than two host plants^18^. The availability of suitable host plants is therefore an a priori requirement for the occurrence of bug herbivore specialists within a given ecosystem.

The positive effects of complex vegetation structure and high plant diversity on insect diversity have been previously documented^19^,^20^,^21^, and specifically bug diversity^22^. Importantly however, the positive effects of complex vegetation structure and high plant diversity may not be general across all insect taxa and functional groups^23^, highlighting the relevance of incorporating trophic- and species-specific responses when investigating the generality of ecological patterns across different ecosystem types.

A mounting body of evidence indicates that managing vegetation structure and plant diversity in urban environments can have positive effects on biodiversity at the landscape level^24^,^25^. Yet, in urban landscapes it is not known which vegetation management actions can promote animal biodiversity in different green space types. Urban green spaces, such as golf courses, public parks and residential gardens, play a crucial role in urban biodiversity conservation^26^,^27^,^28^,^29^,^30^. These urban green spaces, however, are often unintentionally managed and contain a range of both early- and late-succession vegetation features (e.g. turf grass lawns, patches of unmanaged vegetation, trees, shrubs). Understanding how this diversity of habitat structures impacts insects and other animal taxa will inform potential management practices that could promote biodiversity.

In this study we assess the impact of different vegetation management practices on herbivorous and predatory insects by examining heteropteran bug responses to variation in vegetation structure and plant diversity in different urban green space types (golf courses, residential gardens, public parks). Specifically, we use multi-species site occupancy models under a Bayesian mode of inference to:

1. Assess how species richness of herbivorous and predatory bugs varies amongst green space types; and
2. quantify the magnitude of the effect of vegetation volume and plant species diversity on bug trophic (i.e., herbivorous or predatory) and species-specific occupancy.

We also examined the role that habitat area has on bug diversity by assessing the fit of the data to the power function of species-area relationships^31^. The results suggest that area had a positive effect on bug diversity, but was not the key driver of diversity. Instead, vegetation volume and plant diversity across habitat types were the major factors in predicting bug diversity.

## Results

The survey yielded 91 bug species (75 herbivores and 16 predators) from 19 families (Tables S1–S4). This represents approximately 20% of the total bug gamma diversity estimated for Victoria, Australia^32^, and agrees well with the bug species richness found in other temperate urban areas^33^. As many as 98% of all species recorded were native to the study area. Only two species, the green stink bug *Nezara viridula* and the Azalea lacebug *Stephanitis pyrioides*, were non-native to Australia. Of the total number of species, 38 were unique to golf courses whilst only six and eleven were unique to parks and gardens, respectively. Eleven herbivores were observed in all green space types, with the most ubiquitous species being the alydid *Mutusca brevicornis*, occurring in 54% of all plots. By contrast, only two predators were observed in all green space types, with the most ubiquitous species being the Pacific damselbug *Nabis kinbergii*, occurring in 27% of all plots. An assessment of sample completeness showed that further sampling would have resulted in little increase in sample coverage (Fig. S1).

### Species-area model

Area (A) had a positive effect on bug species richness (S), with model estimates indicating that the mean fit of our data to the power function of the species-area relationship followed:


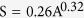


The mean estimate of the intercept parameter *c* (0.26) was associated with a wide 95% credible interval (henceforth CI_95%_) that ranged from 0.08 to 0.64, whereas the mean estimate of the slope parameter *z* (0.32) showed a more accurate CI_95%_ that ranged from 0.23 to 0.40. The observed vs. estimated fit of our data to the power function of the species-area relationship was at best intermediate (r^2^ = 0.43; Fig. S2). There were more observed bug species than those expected by the modelled species-area relationship in approximately 40% of golf course and park sites, while in as much as 90% of garden sites there were less observed species than those predicted by the model.

### Green space type model

The mean probability of occurrence for bugs was high in all green space types, with species estimated to occur at 62% to 86% of sites. By contrast, the mean probability of detection was low, with species estimated to be observed only 4% to 13% of the times when they were present. These trends were consistent across all green space types, as evidenced by the uncertainty associated with the mean responses (Table 1).

**Figure 1:**
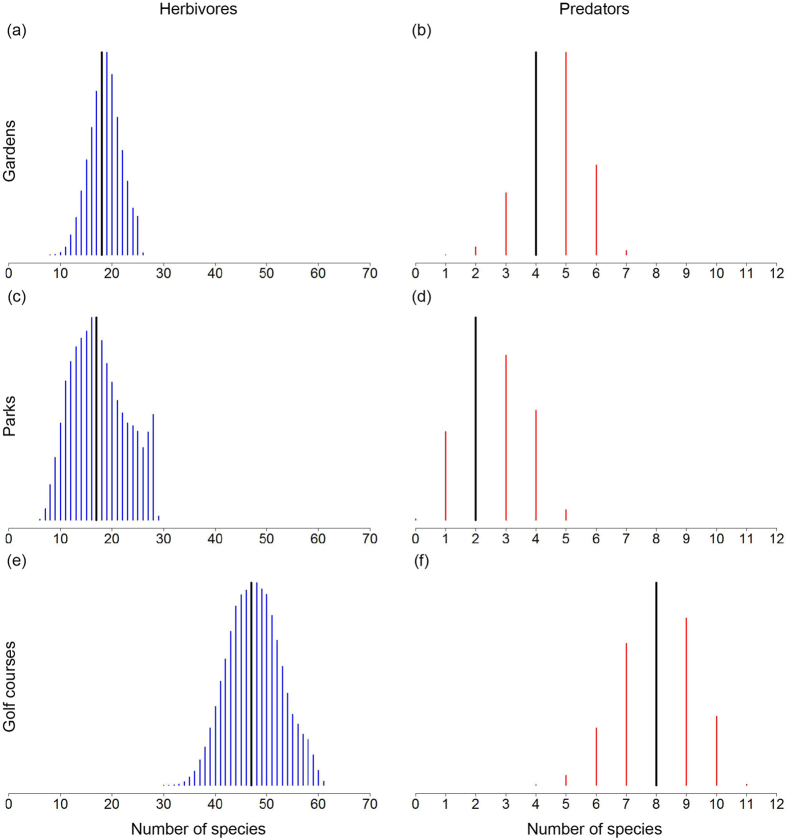
Estimated species richness of herbivorous (**a**,**c**,**e**) and predatory (**b**,**d**,**f**) bugs in gardens (**a**,**b**), parks (**c**,**d**) and golf courses (**e**,**f**). Black lines indicate the mean response and coloured lines the posterior distribution (i.e., 100% credible interval).

The mean bug species richness was substantially higher in golf courses (57 spp.) than in parks (21 spp.) and gardens (24 spp.). The golf course estimate was associated with a CI_95%_ that did not overlap the CIs_95%_ of either parks or gardens (Table 1). The mean estimated species richness for herbivores was higher in golf courses (48 spp.) than in parks (18 spp.) and gardens (19 spp.) (Fig. 1), with the golf course CI_95%_ not overlapping the CIs_95%_ of either parks or gardens (Table 1). Mean estimated species richness for predators was higher in golf courses (9 spp.) than in parks (3 spp.) and gardens (5 spp.) (Fig. 1), with the golf course CI_95%_ not overlapping the parks CI_95%_ but slightly overlapping the gardens CI_95%_ (Table 2). Posterior estimates for the mean, standard deviation and CI_95%_ for the species-specific probabilities of occurrence and detection for each green space are given in Table S1.

### Trophic-level model

The mean probabilities of occurrence for herbivorous and predatory bugs were moderately high, with herbivores and predators estimated to occur on average at 56% and 71% of sites, respectively. In contrast, mean probabilities of detection were low for both trophic groups, with bug species estimated to be observed on average only 3% (predators) to 4% (herbivores) of the times when they were present. These trends were consistent across all green space types, as evidenced by the uncertainty associated with the mean responses (Table 2).

The species-specific probabilities of occurrence for herbivores varied considerably, with individual species estimated to occur in as few as 9% of sites (*Cuspicona sp. 2*) and in as many as 96% of sites (*Nysius caledoniae*). In contrast, the species-specific probabilities of occurrence for predators varied only moderately, with individual species estimated to occur between 54% (*Dicrotelus prolixus*) and 94% (*N. kinbergii*) of sites.

The species-specific probabilities of detection for herbivores also varied considerably, with individual species estimated to be observed between 1% (*N. caledoniae*) and 70% (*Melanocanthus scutellaris*) of the times when they were present. On the other hand, the variation in the species-specific probabilities of detection for predators was much less pronounced, with species estimated to be observed between 1% (*D. prolixus*) and 29% (*Gminatus australis*) of the times when they were present. Posterior estimates for the mean, standard deviation and CI_95%_ for the species-specific probabilities of occurrence and detection for herbivores and predators are given in Table S2.

### Trophic-level effects of covariates

The mean effect of vegetation volume on the probability of occurrence for herbivorous and predatory bugs was positive, with posterior CIs_95%_ that either contained only positive values (herbivores) or with values that slightly overlapped zero (predators) (Table 2). In contrast, the mean effect of plant species diversity was negative for herbivorous bugs and positive for predatory bugs, with posterior CIs_95%_ that distinctly overlapped zero (Table 2).

**Figure 2:**
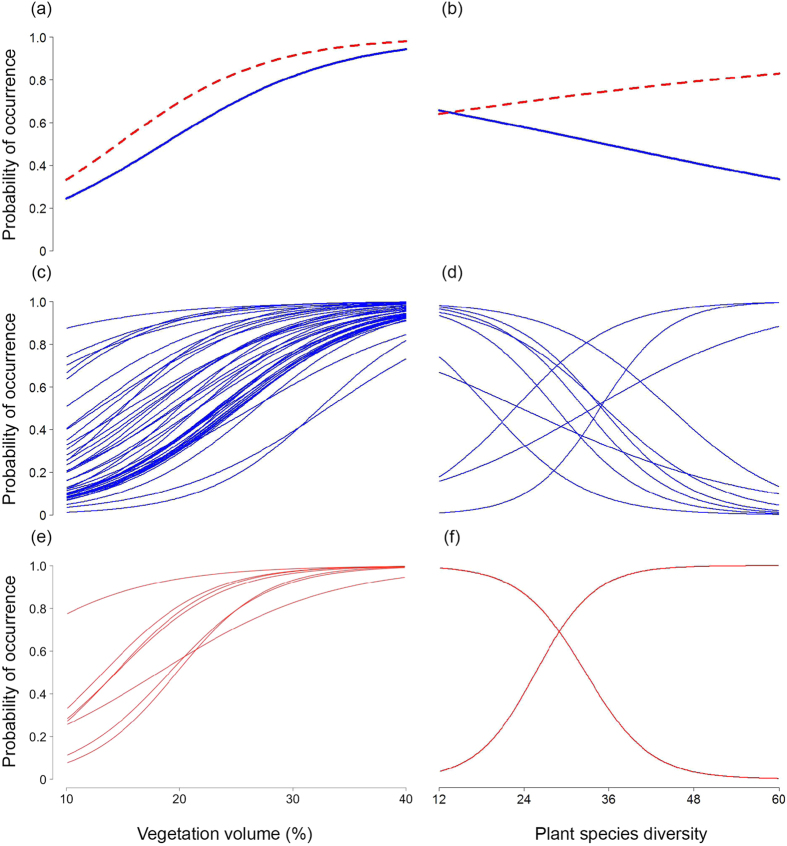
Predicted mean trophic-level (**a**,**b**) and species-specific (**c**–**f**) responses of herbivorous (**a**–**d** blue solid lines) and predatory (**a**,**b**: red dashed lines; (**e**,**f)** red solid lines) bugs to the vegetation volume (**a**,**c**,**d**) and plant species diversity (**b**,**d**,**f**) gradients. Species illustrated are limited to those that showed a strong response to the covariates (i.e., those with 99, 95 and 75% CIs that did not overlap zero).

**Figure 3:**
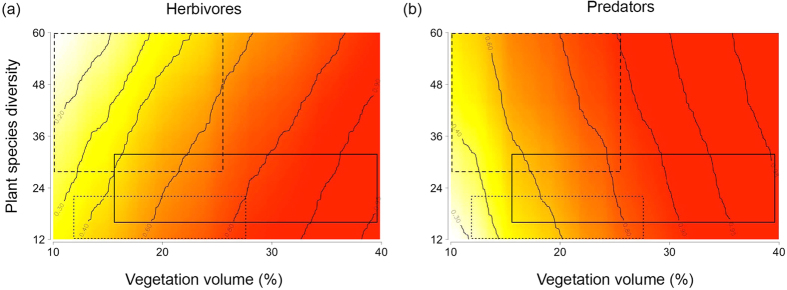
Predicted combined effects of vegetation volume and plant species diversity on the occupancy of herbivorous (**a**) and predatory (**b**) bugs. The superimposed rectangles represent the environmental space defined by the vegetation volume and plant species diversity data points as quantified in each green space type (golf courses: solid line; gardens: dashed line; parks: dotted line).

Predicted data derived from these effects showed that the predictive curves for the mean bug trophic-level response to the vegetation volume gradient had positive slopes for both herbivores and predators (Fig. 2a), whereas the bug trophic-level response to plant species diversity was negative for herbivores and positive for predators (Fig. 2b). When vegetation volume and plant species diversity were combined into a single environmental space, a trend of high occupancy of herbivores was predicted for increasing levels of vegetation volume coupled with decreasing levels of plant species diversity (Fig. 3a). On the other hand, high occupancy of predators was predicted for increasing levels of vegetation volume and plant species diversity (Fig. 3b). In our study, high occupancy levels of herbivores and predators (species occurring in more than 80% of sites) were almost exclusively associated with the environmental space bounding the golf courses’ data points (solid rectangle in Fig. 3a,b). However, high occupancy levels of predators were also associated to an extent with the environmental space bounding the gardens’ data points (dashed rectangle in Fig. 3b), which reflects the strong positive relationship between plant species diversity and occupancy by predators (Fig. 2b).

### Species-specific effects of covariates

The mean species-specific effects of vegetation volume on the probabilities of occurrence for bugs were all positive, varying from 0.010 (*S. pyrioides*) to 1.682 (*M. brevicornis*) in herbivores, and from 0.137 (*Oechalia schellenbergii*) to 1.994 (*D. prolixus*) in predators. In contrast, the mean species-specific effects of plant species diversity were both positive and negative, varying from −2.112 (*Dindymus versicolor*) to 2.702 (*S. pyrioides*) in predators, and from −2.868 (*Orius sp.*) to 3.169 (*Chinoneides tasmaniensis*).

Mean and standard deviation posterior estimates for the covariates’ species-specific effects on the probabilities of occurrence for each herbivorous and predatory bug species are given in Tables S3–S4. We also provide in Tables S3–S4 the posterior estimates for a series of quantiles, from which it is possible to derive a range of credible intervals (99, 95, 75, 50, 25, 5 and 1%) to assess which species had the strongest responses to each of the explanatory variables. We associated species with the three highest CIs (99, 95 and 75%) that did not overlap zero as having a strong response to the given explanatory variable. As much as 59% (44 spp.) of herbivores showed strong positive responses to the vegetation volume gradient (Fig. 2c). The species with the strongest positive responses were *M. brevicornis* and *Stenophyella macreta*, both with CIs_99%_ that contained only positive values. Likewise, as much as 44% (7 spp.) of predators showed strong positive responses to vegetation volume (Fig. 2e), all with CI_75%_ that contained only positive values. Neither herbivores nor predators showed strong negative responses to vegetation volume.

Responses to the plant diversity gradient were more complex, with different species showing either a positive or a negative response. As few as 4% (3 spp.) of herbivores showed a strong positive response to plant species diversity (Fig. 2d). The species showing the strongest positive response was *S. pyrioides*, its CI_99%_ containing only positive values. On the other hand, 9% (7 spp.) of herbivores showed a strong negative response to plant species diversity (Fig. 2d), all with CI_75%_ that contained only negative values. Only two predators showed strong responses to the plant species diversity gradient (Fig. 2f). These species were *C. tasmaniensis* and *Orius sp.*, which showed positive and negative responses, respectively. Both species showed CI_75%_ that did not overlap zero.

## Discussion

Our study demonstrates that there are significant differences in the capacity of different green space types to support diverse herbivorous and predatory bug communities. Vegetation structure has a positive effect on bug diversity at the trophic- and species-specific levels (Fig. 2a); while plant species diversity has a more variable effect, generally increasing the diversity of predators, while reducing the diversity of herbivorous bugs (Fig. 2b). However, in both cases, these general responses to plant diversity are highly species specific, with individual species within each trophic group displaying quite different responses to plant diversity. These results indicate that the changes to urban vegetation associated with different green space management practises, will have a distinct effect on the predatory and herbivorous insects within these urban ecological networks.

In our examination of how bug species richness varied amongst green space types, we find strong evidence that golf courses are likely to sustain more herbivorous and predatory species than parks and gardens (Fig. 1). This finding is consistent with previous studies^27^. Given that in our study golf courses were the largest sites in surface area, this finding is also consistent with the species-area relationship. In our study, however, as much as 40% of golf course sites had more bug species than those predicted by the species-area relationship (Fig. S2). One potential explanation for golf courses presenting higher levels of herbivore and predatory bug species richness is that the diversity of vegetation on golf courses provides a wider range of resource-rich habitats relative to parks and gardens. For example, patches of low-intensity managed or unmanaged vegetation typical of tall grass or ‘rough’ areas of a golf course that contain ruderal grass and forb species may provide resources that support bug assemblages including granivores and grass specialists. Interestingly, analogous ruderal habitats in both agricultural (e.g. oldfields) and urban (e.g. brownfields, derelict sites, vacant lots) environments have been documented to be rich in rare and endangered insect biodiversity^33^,^34^. Additionally, in a parallel study the same golf course plots used in this study had higher native plant species richness than residential and park plots^35^, further supporting our findings that increases in the proportion of native plants may also benefit herbivore and predatory bugs in this system.

Our study strengthens this understanding about the role of golf courses in supporting urban biodiversity in two important ways. Firstly, our study demonstrates that vegetation structure and plant species diversity drive bug diversity, regardless of functional group (e.g. herbivores and predators). Secondly, by examining heteropteran bugs, our study extends our understanding of urban insects to those that show incomplete metamorphosis. Insects are divided into two clades, those that show incomplete metamorphosis, such as heteropteran bugs, and those that show complete metamorphosis, such as butterflies, bumble bees and ground beetles. Species that show incomplete metamorphosis are reliant on similar resources throughout their life, and thus better reflect local environmental conditions than species that vary their resource requirements according to life stage. Future research should focus on elucidating which vegetation features, or which plant species or group of species, contributes most to increases in the biodiversity of herbivorous and predatory insect species in different green space types.

When examined at the trophic-level both herbivorous and predatory bug assemblages show positive responses to vegetation volume (Fig. 2a). Both herbivorous and predatory bug assemblages require vegetation-derived resources to complete their life cycles. Herbivorous bugs, however, interact with vegetation resources directly, utilising their piercing-sucking mouth specialisations to feed on the most nutritional-rich portions of plants such as leaves, pollen, nectar, flower and leaf buds, and seed^36^. Most bug predators on the other hand interact with plant resources indirectly, utilising vegetation elements as hunting and mating grounds. Most interestingly, a few species, in the absence of suitable prey, will secure moisture and supplement their diets by feeding directly from plant resources^36^. These differences are likely to explain why the trophic-level response of herbivores was strictly positive, while the response of predators also included a negative component (Table 2). Vegetation volume also has a strong positive effect on the species-specific probabilities of occurrence of most herbivorous and predatory bug species, with many of the bugs in this study area predicted only to occur in sites with considerable vegetation volume (Fig. 2c,e). Our findings concur with non-urban studies that have investigated the positive relationship between vegetation structure and insect diversity^20^,^22^. However, our study shows that for both herbivorous and predatory insect species this relationship is species-specific, with most, but not all, species showing strong responses to an increase in vegetation volume. For example, most herbivorous and predatory bugs belonging in families traditionally associated with large-bodied species (e.g. Alydidae, Coreidae, Pentatomidae and Reduviidae) show strong positive responses to vegetation volume, indicating perhaps that some of these species have evolved in close association with large amounts of plant resources (e.g. the coreid *Amorbus sp.*, a herbivore closely associated with native Eucalyptus trees^37^). On the other hand, herbivorous bugs belonging in families traditionally associated with small-bodied species with strong degrees of host specificity (e.g. Miridae and Tingidae)^18^ show high site occupancy regardless of the amount of vegetation volume, suggesting that for these species host-specificity is a more important driver of site occupancy than vegetation structure.

From a conservation standpoint, these findings are both concerning and exciting. They imply that most bug species will tend to decline in urban areas if the vegetation structure of green spaces is simplified, for example, if green spaces are managed predominantly as lawns or tree canopies. This simplification process could potentially lead to local extinctions, if it were to occur over large areas. Yet, our findings also imply that small-scale management actions that increase the structure of urban vegetation undertaken by both public (e.g. local governments) and private (e.g. homeowners) actors may encourage a greater diversity of heteropteran bugs. Local governments, for example, could boost insect diversity in their municipality by increasing the amount of mid-storey and grassland-type vegetation, and promote the retention or planting of native plant species.

Our data do not indicate an effect of plant species diversity on herbivorous or predatory bugs at the trophic-level. Rather, our results show that this effect is complex and distinctly species-specific, with species predicted to exhibit both positive and negative responses (Fig. 2d,f). Although bug herbivorous and predatory assemblages are structured by a mix of specialists and generalists, most bug species, particularly predators, tend towards a generalist diet, feeding on a wide array of host plants and arthropod prey^15^,^36^. Consequently, as our results have shown, we will expect that the overall effect of plant diversity on bug communities should be small and that the magnitude of this effect should be strong in only a small proportion of specialist species. For example, amongst the herbivorous species showing a mean positive response to plant species diversity, less than 5% showed a strong response, with the species showing the strongest response being the specialist non-native Azalea lacebug *S. pyrioides*. Azalea lacebugs, as their name implies, show strong host-specificity towards *Rhododendron* (Ericaceae), a genus that in our study occurred exclusively in association with garden sites characterised by high levels of exotic plant diversity^35^. Amongst the predators, only the stilt bug *C. tasmaniensis* showed a strong response to plant species diversity. Interestingly, this species is a specialist predator, feeding exclusively on insect specialist herbivores closely associated with a few native and non-native Geraniaceae. Positive relationships between plant and insect diversity have been recorded in mechanistic experiments by Haddad *et al*.^19^,^20^. However, our study suggests that in urban environments with much higher plant species richness and variability in plant species composition, the relationship between plant diversity and the response of insect species and trophic groups is much less consistent, and may be more strongly related with plant species identity than richness per se.

A potential explanation for this seemingly contradictory result is that in most urban environments, including our study area, high plant diversity is predominantly associated with residential gardens. Overall, residential gardens are thought to contribute substantially to insect diversity^28^, however, many management activities (e.g. pesticide use and high-intensity management) can greatly reduce their contribution^38^. In this study, for example, gardens supported significantly lower bug species richness than the other green space types (Fig. 1), perhaps due to their lower vegetation structural complexity. Alternatively, the high diversity of plant species in many residential gardens, resulting primarily from idiosyncratic human preferences^39^, mean that they may be dominated by non-native species^35^, which may not provide native insect specialists with the resources they need to thrive outside of their natural non-urban ranges. The question of why plant-diverse gardens in urban environments do not attain the high levels of herbivorous and predatory insect species predicted by the non-urban literature remains to be fully explored.

Our results suggest that high bug occupancy can be obtained in green spaces with specific combinations of vegetation structure and plant diversity. When the vegetation volume and plant species diversity gradients were combined into a single predicted environmental space, high occupancy of herbivores and predators was almost exclusively associated with the environmental space bounding the data points of golf courses (Fig. 3). We therefore conclude that large green spaces, such as golf courses, are more likely to support diverse herbivorous and predatory insect assemblages due to their ability to provide a greater heterogeneity of vegetation structure and plant diversity and favourable combinations of these green space attributes. The challenge we now face is understanding how we can boost the conservation value of all urban green spaces for herbivorous and predatory insects through management strategies and actions aimed at promoting synergistic combinations of vegetation structure and plant diversity. This will be especially important in large green spaces with simple vegetation structure, and in smaller green spaces such as public parks and residential gardens where it may be more difficult to intentionally achieve a heterogeneous mix of vegetation structure and diversity. Ultimately, tackling this conservation challenge could provide enormous benefits for all other elements of urban ecological networks, including human city-dwellers.

## Methods

### Experimental design

**Figure 4:**
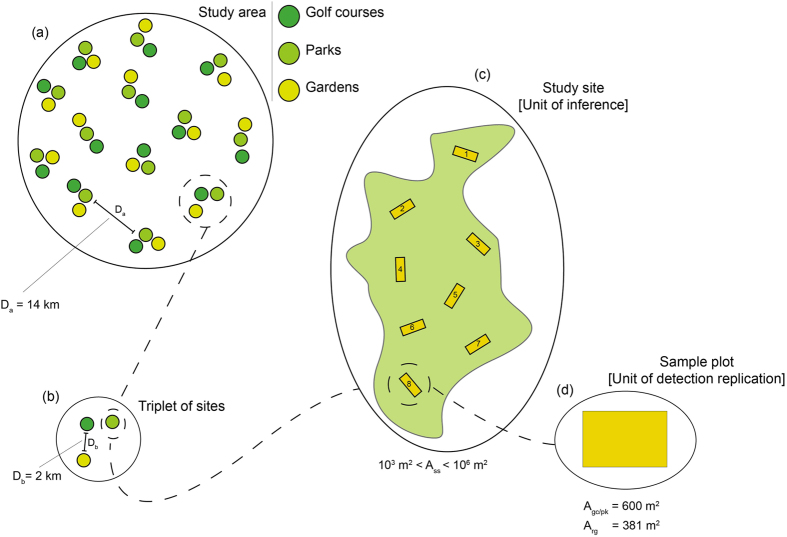
Schematic representation of our experimental design. Within the study area (**a**) we selected 13 triplet of sites (**b**), each of which consisted of a golf course, a park and a garden study site. In total, we surveyed 39 study sites (**c**), which represented the units of inference. Within each site we located a series of sample plots (**d**), which represented the units of detection replication. The figure further indicates the average distance between triplets of sites (D_a_) and study sites (D_b_), as well as the range of area values shown by the study sites (A_ss_) and the average area value for plots within golf course and park (A_gc/pk_) and garden (A_rg_) sites.

The study was conducted in Melbourne, Australia’s second most populated city (4 million inhabitants estimated for 2015) that supports diverse indigenous and introduced biodiversity. Melbourne spans several bioregions, so to standardise underlying geology, climate and remnant vegetation associations we limited the study area (Fig. 4a) to the south-eastern suburbs within the Gippsland Plain bioregion. The bioregion is characterised by sandy soils, average monthly maximum temperatures 13.5–25.9 °C, and average monthly rainfall 47.3–66.1 mm. The dominant native vegetation communities are grassy woodland and heathy woodland with a eucalypt overstorey^40^. The main types of urban green space include public parks, gardens, golf courses and scattered patches of remnant native vegetation^41^.

Within the study area, we mapped all golf courses and identified all residential neighbourhoods and public parks surrounding each golf course. We then identified triplets of green space sites (golf course, residential gardens, public park; Fig. 4b) established at approximately the same time (e.g. decade) as determined from historical aerial imagery and municipal land release records. In all, 39 sites (Fig. 4c) were randomly selected and stratified by the three green space types. In our study, these sites constituted the units of inference – that is, the spatial sample units in which we collected data to draw inferences on the ecological process (i.e. species site occupancy)^42^. Patches of remnant vegetation were not included in the study as they are unevenly-distributed and our study is focused on the effects of vegetation management in ‘human constructed’ green spaces. Triplets of sites were located on average 14 km from each other, and sites within each triplet were located at least two km from each other. This configuration was designed to generate a spatial distribution of sites that fulfills the requirement that observations should be drawn from spatially-independent units (i.e. each observation brings one full degree of freedom^43^). We therefore expected no positive effects of spatial-autocorrelation associated with our inferences. Site area ranged approximately across four orders of magnitude, varying from 6,712 to 862,022 m^2^ (mean = 273,193 m^2^).

Within each site we placed a series of sample plots (Fig. 4d), which in our study constituted the unit of detection replication – that is, the sample spatial units in which we collected data to draw inferences on the observation process (i.e. species detectability)^42^. We placed a minimum of two plots in sites < 50,000 m^2^ in size, with two additional plots being placed for every 50,000 m^2^ increase in site size. This configuration yielded a maximum of eight plots in golf courses (for details of plot placements see Threlfall *et al*.^29^). Taken together, we collected samples in 182 plots: 104 in golf course, 52 in residential gardens and 26 in public parks. Within each golf courses we placed half of the plots in ‘woodland rough’, characterised by low-intensity managed shrub and tree vegetation, and the other half in ‘long grassy rough’, characterised by low-intensity managed or unmanaged herbaceous vegetation without trees. Golf course and park plots had an area of 600 m^2^ (20 × 30 m), while garden plots ranged from 211 to 870 m^2^ (mean = 381 m^2^).

### Insect sampling, sorting and identification

Insects were collected from 14 January to 12 March 2012 (Australian summer). At each plot, insects were collected from aboveground vegetation with 200 sweeps of an entomological net (50 cm diameter), transferred to 70% ethanol-filled containers for storage and preservation, and posteriorly sorted to order, and, whenever possible, bugs identified to species. Unlike many other insect taxa, bugs are taxonomically tractable, allowing specimens to be sorted to morphospecies and often to named species.

### Explanatory variables

To measure vegetation structure within a plot, four parallel transects were established and sampled at 5 m intervals. At each interval, we recorded the identity and growth form of any plant species that intercepted a 2.5 m high pole at five height intervals (0.0–0.2 m; 0.2–0.5 m; 0.5–1.0 m; 1.0–2.0 m; >2.0 m). These data were used to calculate the volume of vegetation within a given height band. To account for the variable sizes of garden plots, we divided these volumes by the total available volume (i.e., area sampled multiplied by height of relevant sampling interval) to generate a variable of vegetation volume (*vvol*: percentage of volume occupied by vegetation). Additionally, the species identity data were used to generate a variable of plant diversity (*psd*: plant species diversity). Vegetation variables at the plot level were then used to calculate the average for a given green space site via averaging the values recorded for plots within each green space. In our study, *vvol* ranged from < 1% to 40% (mean = 20%), and *psd* from 12 to 70 plant species per plot (mean = 27).

### Modelling framework: Multi-species site occupancy models

Multi-species site occupancy models or community occupancy models are grounded in the idea that communities and metacommunities can be described as a collection of individual species^42^,^44^. The hierarchical structure of multi-species site occupancy models is composed of three levels: a level for the ecological process (e.g. species site occupancy), another for the observation process (i.e. species detectability), and a third to account for the sampling of each species from its (meta)community. The model is therefore a (meta)community *hypermodel*, in which the occupancy, detection and effects parameters for each species are treated as random effects governed by *hyperparameters* that describe the (meta)community^42^.

A key advantage of multi-species site occupancy models is that they allow inferences at both the species level, such as the effects of covariates on the occupancy probability of each individual species, and community level, such as community responses to random effects (e.g. green space type or trophic-level). Another key value of this modelling framework is that the observation process hierarchical level reduces or even eliminates the bias generated by the imperfect detection of species^45^,^46^. Treating each species as random effects is yet another key feature of multi-species site occupancy models, particularly as this approach allows for the estimation of the species richness for the whole observed community, as well as the number of species occurring at each specified community level random effect (e.g. site, green space type, trophic-level)^42^,^44^. From the conservation point of view, multi-species site occupancy models are flexible analytical tools with the potential to improve assessments of biodiversity responses to management-oriented actions^47^.

### Statistical analyses

We analysed our data using two variations of the multi-species site occupancy models provided by Zipkin *et al*.^48^ and Mata *et al*.^33^. In both models we confidently assumed that the species pool remained constant throughout the study, satisfying therefore an important assumption of the modelling framework.

Our models included an extra hierarchical level that specified that the species-level random effects were governed by urban green space type (*UGS-model*) or trophic-level (*TL-model*) hyperparameters. The occurrence model was specified as:


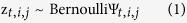


where Ψ_*t*,*i*,*j*_ is the probability that, within green space type (*UGS-model*) or trophic-level (*TL-model*) *t*, species *i* occurs at site *j*, and the detection model as:


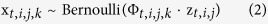


where Φ_*t*,*i*,*j*,*k*_ is, within green space type (*UGS-model*) or trophic-level (*TL-model*) *t*, the detection probability of species *i* at site *j* at plot *k*. This specification satisfies the condition that the probability of detecting a species will be zero when it is not present.

In the *UGS-model*, the linear predictor of the occupancy model on the logit-probability scale was specified as:


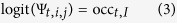


while in the *TL-model* the occupancy model linear predictor was specified as:


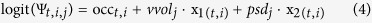


where Ψ_*t*,*i*,*j*_ are the species-specific probabilities of occurrence for green space type (*UGS-model*) or trophic-level (*TL-model*) *t*; occ_*t*,*i*_ the species-level random effects for green space type (*UGS-model*) or trophic-level (*TL-model*) *t*; x_1(*t*,*i*)_ and x_2(*t*,*i*)_ the effects of covariates on the species-specific occurrence probabilities for trophic-level *t*; and *vvol*_*j*_ and *psd*_*j*_ the *mean* = 0, *sd* = 1 standardised values for the vegetation volume (*vvol*) and plant species diversity (*psd*) covariates for each site *j*. In the *UGS-model*, the species-level random effects occ_*t*,*i*_ were specified as:


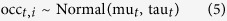


where mu_*t*_ ~ log(omega) − log(1 − omega) and tau_*t*_ ~ Gamma (0.1, 0.1). Thus, the green space type occupancy hyperparameters were also considered random-effects governed by the global occupancy hyperparameter omega ~ Uniform (0, 1).

In the *LP-model*, the species-level random effects occ_*t*,*i*_ were specified as:


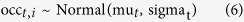


where mu_*t*_ ~ Normal (mu, sigma) and sigma_*t*_ ~ Cauchy (0, 2.5). Thus, the trophic-level occupancy hyperparameters were also considered random-effects governed by the global occupancy hyperparameters mu ~ Cauchy (0, 2.5) and sigma ~ Cauchy (0, 2.5). This specification of normally-distributed hyperparameters with weakly informative Cauchy (0, 2.5) priors follows Gelman *et al*.^49^ and Stan Development Team^50^.

The effects of the covariates on species-specific occupancy x_1(*t*,*i*)_ and x_2(*t*,*i*)_ (*TL-model*) were specified as:


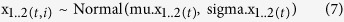


where mu.x_1..2(*t*)_ ~ Normal (mu.x_1..2_, sigma.x_1..2_) and sigma.x_1..2(*t*)_ ~ Cauchy (0, 2.5). Thus, the trophic-level effect hyperparameters were governed by the global effect hyperparameters mu.x_1..2_ ~ Cauchy (0, 2.5) and sigma.x_1..2_ ~ Cauchy (0, 2.5). Finally, we assumed in both the *UGS-* and *TL-model* that the detection probability of species *i* did not vary based on any measured covariate, and was thus determined by an unspecified species-level effect det_*t*,*i*_ as:


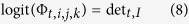


In the *UGS-model*, we estimated the total species richness of each green space type *t*, as well as the species richness of herbivorous and predatory species, using the following summation structure:


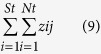


where, within each green space *t, S*_*t*_ is the total number of sites, *N*_*t*_ the total number of detected species, and z_*i*,*j*_ the latent occurrence matrix. As these calculations were done as derived quantities within our Bayesian modelling framework, we were able to report the species richness estimations with their full associated uncertainties.

### Predictions

Using the model’s trophic-level hyperparameters, we predicted trophic-level occupancy for herbivores and predators for 500 values within the range of the vegetation volume (*vvol*) and plant species diversity (*psd*) gradients. To guarantee predictions for a reasonable range of the gradients, we removed the 2.5% most extreme values from each end of the *vvol* and *psd* original ranges. We used these predictions to graphically represent (1) the individual effects of *vvol* and *psd* on the occupancy of herbivores and predators (Fig. 2), and (2) the combined effect of *vvol* and *psd* on the occupancy of herbivores and predators (Fig. 3). Into this latter predicted environmental space, we superimposed three rectangles, which represent the environmental space bounding the data points of the *vvol* and *psd* gradients as quantified in each green space type.

### Species-area model

We modelled the effect of site area on bug species richness using the power function of the species-area relationship^31^:


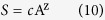


where S equals the number or bug species within a site, A the site’s area, and *c* and *z* are function parameters. In the function’s linearised, logarithmically-transformed version, *c* equals the number of species in one unit of area and *z* the slope of the species-area line.

The model for the power function of the species-area relationship was specified as:


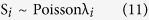


and the non-linear predictor as:


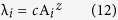


where, in each site *i*, S_*i*_ and A_*i*_ are the number of observed bug species per site and the site’s area, respectively, and λ_*i*_ the intensity parameter, which in a Poisson distribution equals both the mean and variance^42^. The latent variables *c* and *z* were given non-informative Uniform (0, 1) priors.

We used the model’s parameters *c* and *z* to predict the number of bug species per site given the empirical site area data. Using a normally-distributed linear model (function *lm* in the R statistical environment^51^) we correlated these estimations with the observed species richness to obtain a coefficient of determination (r^2^). The value of r^2^ indicated the proportion of the variance in the estimated number of bug species that is predictable from our model – that is, the strength, or lack thereof, by which our data fits the power function of the species-area relationship.

### Bayesian inference implementation

Model parameters were estimated under a Bayesian mode of inference. We used Markov Chain Monte Carlo (MCMC; *Urban green spaces model* and *Species-area relationship model*) and Hamiltonian Monte Carlo (HMC; *Trophic levels model*) simulations to draw samples from the parameters’ posterior distributions. MCMC algorithms were implemented in OpenBUGS^52^/JAGS^53^, accessed through the R packages *R2OpenBUGS*^54^/*jagsUI*^55^. Our MCMC implementation used three chains of 50,000 iterations, discarding the first 5,000 in each chain as burn-in. HMC algorithms were implemented in Stan^56^, accessed through the R package *rstan*^57^. Our HMC implementation used four chains of 5,000 iterations, discarding the first half iterations in each chain during warm-up. Visual inspections of MCMC and HMC chains plus values of the Gelman-Rubin statistic (R-hat < 1.1) indicated acceptable convergence^48^,^58^.

The codes and data to re-run the analyses and generate plots are provided in the Supplementary Information.

## Additional Information

**How to cite this article**: Mata, L. *et al*. Conserving herbivorous and predatory insects in urban green spaces. *Sci. Rep.* **7**, 40970; doi: 10.1038/srep40970 (2017).

**Publisher's note:** Springer Nature remains neutral with regard to jurisdictional claims in published maps and institutional affiliations.

## Supplementary Material

Supplementary Information

Supplementary Dataset 1

Supplementary Dataset 2

Supplementary Dataset 3

Supplementary Dataset 4

Supplementary Dataset 5

Supplementary Dataset 6

Supplementary Dataset 7

## Figures and Tables

**Table 1:** Posterior estimates for the probabilities of occurrence, probabilities of detection and species richness as derived from the urban green space model.

Parameter	Mean	SD	CI 2.5%	CI 97.5%
Probability of occurrence				
Golf courses	0.859	0.087	0.665	0.985
Parks	0.629	0.196	0.299	0.976
Gardens	0.755	0.125	0.492	0.975
Probability of detection				
Golf courses	0.043	0.010	0.026	0.065
Parks	0.137	0.051	0.068	0.262
Gardens	0.077	0.022	0.043	0.130
Species richness				
Total				
Golf courses	56.59	6.03	45.30	68.47
Parks	21.07	6.20	10.81	32.33
Gardens	24.18	3.79	16.76	31.33
Herbivores				
Golf courses	48.10	5.15	38.55	58.17
Parks	18.04	5.26	9.33	27.34
Gardens	19.12	2.99	13.27	24.62
Predators				
Golf courses	8.49	1.16	6.16	10.54
Parks	3.03	1.00	1.34	4.82
Gardens	5.06	0.93	3.18	6.69

SD: Standard deviation; CI: Credible interval.

**Table 2:** Posterior estimates for the probabilities of occurrence, probabilities of detection and effects of the vegetation volume and plant species diversity covariates as derived from the trophic-level model.

Parameter	Mean	SD	CI 2.5%	CI 97.5%
Probability of occurrence				
Herbivores	0.559	0.631	0.334	0.804
Predators	0.709	0.789	0.277	0.984
Probability of detection				
Herbivores	0.037	0.556	0.024	0.056
Predators	0.029	0.601	0.013	0.060
Effect of vegetation volume				
Herbivores	1.032	0.305	0.535	1.738
Predators	1.203	0.921	−0.095	3.610
Effect of plant species diversity				
Herbivores	−0.370	0.404	−1.273	0.365
Predators	0.278	1.060	−1.666	2.667

SD: Standard deviation; CI: Credible interval.
