# Supplementary material for: Conserving herbivorous and predatory insects in urban green spaces
Source: Sci Rep. 2017 Jan 19;7:40970. doi: 10.1038/srep40970 (PMC5244475; doi:10.1038/srep40970)
Supplement: Supplementary Information [file srep40970-s1.pdf]

Supplementary Information (Scientific Reports)

Conserving herbivorous and predatory insects in urban green spaces

Luis Mata[a*], Caragh G. Threlfall[b], Nicholas S.G. Williams[b,c], Amy K. Hahs[c], Mallik Malipatil[d], Nigel E. Stork[e] and Stephen J. Livesley[b].

[a] Interdisciplinary Conservation Science Research Group, School of Global, Urban and Social Studies, RMIT University, Melbourne 3000, Victoria, Australia

[b] School of Ecosystem and Forest Sciences, Faculty of Science, The University of Melbourne, Richmond 3121, Victoria, Australia

[c] Australian Research Centre for Urban Ecology, Royal Botanic Gardens Victoria c/o School of BioSciences, The University of Melbourne, Parkville 3010, Victoria, Australia

[d] Department of Economic Development, Jobs, Transport and Resources, AgriBio, La Trobe University, Bundoora 3083, Victoria, Australia

[e] Environmental Futures Research Institute, Griffith School of Environment, Griffith University, Nathan 4111, Queensland, Australia

*Correspondence: Luis Mata, Interdisciplinary Conservation Science Research Group, School of Global, Urban and Social Studies, RMIT University, 124 La Trobe Street, Melbourne 3000, Victoria, Australia. e-mail: luis.mata@rmit.edu.au Phone: +61 3 9925 9945

Species	Family	Golf courses								Parks								Gardens							
		Occurrence				Detection				Occurrence				Detection				Occurrence				Detection			
		mean	sd	CI 2.5%	CI 97.5%	mean	sd	CI 2.5%	CI 97.5%	mean	sd	CI 2.5%	CI 97.5%	mean	sd	CI 2.5%	CI 97.5%	mean	sd	CI 2.5%	CI 97.5%	mean	sd	CI 2.5%	CI 97.5%
**Herbivores**																									
Amorbus sp.	Coreidae	0.851	0.894	0.171	0.998	0.040	0.678	0.010	0.175	0.723	0.813	0.268	0.980	0.145	0.640	0.055	0.348								
Anischys sp.	Acanthosomatidae	0.862	0.898	0.132	0.999	0.017	0.711	0.003	0.096																
Baclozygum depressum Bergroth, 1909	Thaumastocoridae	0.882	0.876	0.288	0.999	0.070	0.633	0.026	0.189																
Brentiscerus putoni (Buchanan White, 1878)	Rhyparochromidae	0.901	0.886	0.238	0.999	0.025	0.675	0.006	0.096	0.691	0.815	0.222	0.979	0.129	0.648	0.043	0.329	0.754	0.803	0.200	0.989	0.043	0.682	0.009	0.166
Chaetedus longiceps Eyles, 1975	Miridae	0.979	0.826	0.831	1.000	0.657	0.552	0.562	0.742	0.774	0.803	0.354	0.985	0.227	0.625	0.102	0.454	0.874	0.764	0.583	0.991	0.225	0.590	0.124	0.369
Cletus similis (Blöte, 1935)	Coreidae	0.856	0.899	0.131	0.998	0.017	0.712	0.003	0.095																
Coridromius sp.	Miridae	0.950	0.852	0.574	0.999	0.058	0.609	0.024	0.124																
Creontiades dilutus (Stäl, 1859)	Miridae	0.902	0.888	0.245	0.999	0.024	0.676	0.006	0.097																
Crompus oculatus Stäl, 1874	Lygaeidae									0.662	0.823	0.169	0.980	0.110	0.659	0.032	0.307								
Crompus opacus Scudder, 1958	Lygaeidae	0.866	0.897	0.168	0.999	0.016	0.708	0.003	0.087																
Cryptorhamphus orbus Stäl, 1860	Cryptorhamphidae	0.747	0.914	0.065	0.997	0.036	0.732	0.006	0.253																
Cuspicona sp. 1	Pentatomidae	0.866	0.853	0.383	0.997	0.129	0.607	0.061	0.262									0.793	0.793	0.279	0.987	0.056	0.659	0.016	0.177
Cuspicona sp. 2	Pentatomidae	0.692	0.895	0.104	0.996	0.071	0.700	0.017	0.293																
Cymodema basicornis (Motschulsky, 1863)	Cymidae	0.920	0.872	0.337	0.999	0.033	0.651	0.009	0.101																
Dictyotus sp. 1	Pentatomidae	0.872	0.898	0.136	0.999	0.016	0.708	0.003	0.091																
Dictyotus sp. 2	Pentatomidae	0.906	0.882	0.268	0.999	0.024	0.673	0.006	0.093																
Dieuches maculicollis (Walker, 1872)	Rhyparochromidae																	0.765	0.803	0.195	0.984	0.042	0.680	0.009	0.155
Dilompus robustus Scudder, 1957	Artheneidae																	0.787	0.797	0.277	0.988	0.057	0.660	0.016	0.180
Dilompus woodwardi Malipatil, 1988	Artheneidae	0.867	0.899	0.143	0.998	0.017	0.708	0.003	0.088	0.659	0.822	0.175	0.974	0.110	0.660	0.032	0.305								
Dindymus versicolor (Herrich-Schaeffer, 1853)	Pyrrhocoridae	0.931	0.866	0.416	0.999	0.041	0.633	0.014	0.108																
Diplocysta trilobata Drake & Poor, 1939	Tingidae	0.865	0.899	0.137	0.999	0.017	0.713	0.003	0.096																
Eribotes sp.	Pentatomidae	0.900	0.884	0.247	0.999	0.024	0.675	0.006	0.097																
Eritingis sp.	Tingidae	0.867	0.899	0.136	0.999	0.016	0.710	0.003	0.093																
Euander lacertosus (Erichson, 1842)	Rhyparochromidae	0.872	0.899	0.155	0.999	0.016	0.710	0.003	0.091	0.661	0.822	0.175	0.981	0.111	0.660	0.033	0.309								
Eupolemus sp. 1	Acanthosomatidae	0.860	0.898	0.123	0.999	0.017	0.710	0.003	0.096	0.658	0.823	0.169	0.977	0.110	0.659	0.032	0.303								
Eupolemus sp. 2	Acanthosomatidae	0.832	0.896	0.151	0.998	0.041	0.682	0.010	0.185																
Eurynysius sp.	Lygaeidae									0.658	0.823	0.171	0.971	0.110	0.660	0.032	0.305								
Eysarcoris distinctus Schouteden, 1906	Pentatomidae	0.836	0.875	0.251	0.998	0.090	0.639	0.033	0.242	0.656	0.824	0.174	0.988	0.111	0.660	0.032	0.306								
Froggattia olivinia Froggatt, 1901	Tingidae	0.867	0.899	0.138	0.998	0.017	0.711	0.003	0.093	0.645	0.825	0.164	0.972	0.136	0.656	0.045	0.365								
Gelonus tasmanicus (Le Guillou, 1841)	Coreidae	0.867	0.901	0.138	0.999	0.017	0.710	0.003	0.094																
Halticus minutus Reuter, 1885	Miridae	0.903	0.841	0.503	0.998	0.174	0.585	0.098	0.292	0.756	0.805	0.326	0.973	0.210	0.630	0.089	0.440	0.765	0.808	0.200	0.989	0.042	0.681	0.009	0.158
Koscocrompus obscurus Scudder, 1958	Lygaeidae	0.867	0.900	0.135	0.998	0.017	0.710	0.003	0.096																
Lethaeini 1	Rhyparochromidae																	0.758	0.807	0.198	0.986	0.043	0.681	0.009	0.163
Malandiola sp.	Tingidae	0.869	0.896	0.142	0.998	0.017	0.708	0.003	0.091																
Mcateella elongata Hacker, 1927	Piesmatidae	0.951	0.857	0.564	0.999	0.058	0.609	0.024	0.123	0.697	0.822	0.222	0.980	0.128	0.648	0.044	0.325								
Melanacanthus scutellaris (Dallas, 1852)	Alydidae	0.878	0.877	0.281	0.999	0.070	0.634	0.026	0.190																
Mictis profana (Fabricius, 1803)	Coreidae	0.900	0.882	0.243	0.999	0.024	0.675	0.006	0.096	0.657	0.823	0.178	0.957	0.111	0.660	0.033	0.306								
Mirini 1	Miridae	0.961	0.842	0.677	0.999	0.113	0.580	0.062	0.191	0.828	0.777	0.417	0.989	0.088	0.630	0.032	0.210								
Mirini 3	Miridae	0.896	0.886	0.233	0.999	0.025	0.677	0.006	0.097																
Mirini 4	Miridae	0.867	0.901	0.131	0.999	0.017	0.712	0.003	0.097	0.660	0.826	0.170	0.974	0.110	0.660	0.032	0.302								
Mirini 5	Miridae	0.901	0.882	0.247	0.999	0.024	0.674	0.006	0.094	0.713	0.815	0.255	0.972	0.172	0.640	0.069	0.408								
Mirini 7	Miridae	0.863	0.900	0.132	0.998	0.017	0.710	0.003	0.093									0.826	0.780	0.410	0.990	0.088	0.630	0.032	0.210
Mirini 8	Miridae	0.864	0.900	0.125	0.999	0.017	0.711	0.003	0.096									0.760	0.807	0.239	0.986	0.042	0.681	0.010	0.161
Mirini 9	Miridae									0.662	0.827	0.175	0.977	0.109	0.659	0.032	0.301	0.792	0.791	0.309	0.989	0.095	0.641	0.033	0.256
Mutusca brevicornis (Dallas, 1852)	Alydidae	0.979	0.827	0.829	0.999	0.760	0.558	0.671	0.835	0.785	0.802	0.375	0.987	0.273	0.626	0.126	0.519	0.729	0.784	0.274	0.989	0.210	0.643	0.084	0.476
Nezara viridula (Linnaeus, 1758)	Pentatomidae	0.864	0.898	0.130	0.998	0.017	0.711	0.003	0.089																
Notius depressus Dallas, 1851	Pentatomidae									0.658	0.822	0.171	0.984	0.110	0.661	0.032	0.311								
Nysius caledoniae Distant, 1920	Lygaeidae	0.979	0.825	0.831	0.999	0.332	0.551	0.248	0.425	0.694	0.819	0.226	0.977	0.128	0.648	0.043	0.327	0.894	0.761	0.631	0.993	0.287	0.579	0.175	0.427
Nysius vinitor Bergroth, 1891	Lygaeidae	0.963	0.833	0.724	0.999	0.210	0.564	0.137	0.303	0.742	0.806	0.307	0.987	0.165	0.633	0.064	0.374	0.811	0.787	0.366	0.989	0.072	0.644	0.023	0.195
Ocirrhoe sp. 1	Pentatomidae	0.441	0.895	0.038	0.992	0.095	0.758	0.014	0.463									0.763	0.809	0.188	0.987	0.042	0.681	0.009	0.160
Ocirrhoe sp. 2	Pentatomidae	0.866	0.899	0.126	0.999	0.017	0.712	0.003	0.098																
Ocirrhoe sp. 3	Pentatomidae	0.923	0.873	0.334	0.999	0.032	0.650	0.009	0.099	0.663	0.827	0.171	0.973	0.110	0.660	0.033	0.306	0.760	0.807	0.203	0.987	0.042	0.682	0.009	0.167
Oncocoris geniculatus (Dallas, 1851)	Pentatomidae	0.897	0.887	0.241	0.999	0.025	0.677	0.006	0.098																
Ontiscus australis Stäl, 1874	Cymidae	0.896	0.884	0.226	0.999	0.024	0.676	0.006	0.097																
Orthotylinae 2	Miridae	0.861	0.895	0.159	0.998	0.017	0.709	0.003	0.093																
Orthotylinae 3	Miridae	0.870	0.900	0.150	0.999	0.017	0.710	0.003	0.093																
Orthotylinae 7	Miridae									0.657	0.824	0.205	0.985	0.110	0.660	0.032	0.308								
Oxycarenus (Oxycarenus) arctatus (Walker, 1872)	Oxycarenidae	0.864	0.902	0.128	0.998	0.017	0.711	0.003	0.098																
Oxycarenus (Oxycarenus) luctuosus (Montrouzier, 1861)	Oxycarenidae	0.868	0.899	0.120	0.999	0.016	0.711	0.003	0.094									0.764	0.807	0.202	0.990	0.042	0.681	0.009	0.161
Pentatomidae 1	Pentatomidae	0.707	0.894	0.105	0.997	0.070	0.699	0.017	0.294	0.656	0.825	0.169	0.972	0.111	0.660	0.033	0.308								
Pentatomidae 20	Pentatomidae									0.661	0.827	0.175	0.969	0.110	0.660	0.032	0.303								
Phylinae 1	Miridae	0.847	0.896	0.165	0.998	0.040	0.680	0.010	0.173	0.655	0.822	0.171	0.970	0.111	0.661	0.032	0.307	0.790	0.793	0.279	0.986	0.057	0.658	0.016	0.176
Phylinae 7	Miridae	0.755	0.911	0.066	0.997	0.035	0.726	0.007	0.240																
Plinthisus (Locutius) woodwardi Slater & Sweet, 1977	Rhyparochromidae	0.863	0.899	0.126	0.999	0.017	0.711	0.003	0.096																
Plinthisus sp. 2	Rhyparochromidae																	0.759	0.807	0.189	0.983	0.042	0.680	0.009	0.165
Remaudiereana inornata (Walker, 1872)	Rhyparochromidae	0.979	0.824	0.829	0.999	0.399	0.550	0.310	0.496	0.743	0.807	0.307	0.983	0.165	0.632	0.066	0.368	0.846	0.766	0.520	0.992	0.260	0.596	0.143	0.430
Sidnia kinbergi (Stäl, 1859)	Miridae	0.972	0.831	0.782	0.999	0.234	0.559	0.159	0.324	0.696	0.819	0.226	0.986	0.128	0.648	0.043	0.327	0.773	0.776	0.354	0.987	0.212	0.625	0.094	0.432
Stenophyella macreta Horváth, 1914	Pachygronthidae	0.882	0.710	0.640	0.981	0.489	0.555	0.382	0.594	0.640	0.824	0.160	0.963	0.137	0.658	0.044	0.373	0.755	0.803	0.189	0.986	0.042	0.683	0.009	0.163
Stephanitis pyroides (Scott, 1874)	Tingidae																	0.848	0.775	0.488	0.992	0.141	0.609	0.064	0.280
Stizocephalus hirsutus Scudder, 1975	Rhyparochromidae	0.862	0.897	0.141	0.998	0.017	0.711	0.003	0.093																
Thaumastocoris safordi Noack, Cassis & Rose, 2011	Thaumastocoridae	0.855	0.902	0.123	0.998	0.017	0.711	0.003	0.101	0.661	0.829	0.169	0.980	0.110	0.660	0.031	0.308								
Tingis sp. 1	Tingidae	0.918	0.875	0.336	0.999	0.033	0.652	0.009	0.102									0.809	0.787	0.345	0.991	0.072	0.644	0.023	0.196
Tingis sp. 2	Tingidae																	0.765	0.804	0.210	0.990	0.042	0.680	0.009	0.156
Ulonemia sp.	Tingidae																	0.760	0.803	0.199	0.986	0.042	0.680	0.009	0.161
Zanessa rubrovariegata Kirkaldy, 1902	Miridae	0.871	0.895	0.142	0.998	0.016	0.709	0.003	0.090																
**Predators**																									
Buchananiella whitei Reuter, 1884	Anthocoridae	0.863	0.902	0.131	0.999	0.017	0.713	0.003	0.099																
Cermatulus nasalis nasalis (Westwood, 1837)	Pentatomidae	0.919	0.874	0.327	0.998	0.033	0.652	0.009	0.104																
Chinoneides tasmaniensis (Gross, 1950)	Berytidae	0.922	0.875	0.334	0.999	0.033	0.650	0.009	0.099									0.787	0.770	0.399	0.986	0.252	0.615	0.123	0.464
Coranus callosus Stäl, 1874	Reduviidae	0.898	0.886	0.238	0.999	0.025	0.675	0.006	0.097																
Deraeocorini 1	Miridae																	0.763	0.805	0.201	0.987	0.042	0.680	0.009	0.160
Deraeocorini 2	Miridae																	0.751	0.912	0.068	0.998	0.036	0.728	0.006	0.248
Dicrotelus prolixus Erichson, 1842	Reduviidae	0.841	0.893	0.157	0.998	0.040	0.678	0.010	0.172																
Dicyphini 1	Miridae																	0.763	0.804	0.209	0.987	0.042	0.681	0.009	0.156
Dicyphini 2	Miridae																	0.761	0.806	0.198	0.987	0.042	0.682	0.009	0.163
Emesinae 1	Reduviidae	0.822	0.896	0.147	0.998	0.042	0.684	0.010	0.188	0.657	0.824	0.173	0.970	0.110	0.660	0.033	0.310	0.790	0.792	0.289	0.987	0.057	0.659	0.016	0.179
Germalus victoriae Bergroth, 1895	Geocoridae									0.660	0.827	0.173	0.971	0.110	0.659	0.032	0.305								
Gminatus australis (Erichson, 1842)	Reduviidae	0.898	0.882	0.264	0.999	0.025	0.674	0.006	0.096	0.657	0.825	0.173	0.973	0.111	0.660	0.032	0.308								
Nabis (Tropiconabis) kinbergii Reuter, 1872	Nabidae	0.979	0.824	0.830	0.999	0.362	0.551	0.275	0.457	0.744	0.807	0.307	0.987	0.165	0.633	0.067	0.366	0.855	0.770	0.512	0.989	0.118	0.613	0.050	0.243
Oechalia schellenbergii (Guérin, 1831)	Pentatomidae																	0.760	0.808	0.193	0.990	0.043	0.682	0.009	0.163
Orius sp.	Anthocoridae	0.847	0.864	0.310	0.997	0.111	0.621	0.048	0.255	0.658	0.823	0.175	0.951	0.110	0.658	0.032	0.300								
Stylogeocoris biroi Montandon, 1913	Geocoridae	0.865	0.897	0.145	0.999	0.016	0.710	0.003	0.088																

Table S1. Posterior estimates of the species-specific probabilities of occurrence and detection of heteropteran bugs as estimated under the urban green space types model.

Species	Family	Occupancy				Detection			
		mean	sd	CI2.5	CI97.5	mean	sd	CI2.5	CI97.5
**Herbivores**									
Amorbus sp.	Coreidae	0.243	0.811	0.024	0.898	0.085	0.659	0.026	0.250
Anischys sp.	Acanthosomatidae	0.379	0.876	0.019	0.976	0.016	0.707	0.003	0.086
Baclozygum depressum Bergroth, 1909	Thaumastocoridae	0.438	0.805	0.072	0.955	0.063	0.635	0.022	0.164
Brentiscerus putoni (Buchanan White, 1878)	Rhyparochromidae	0.721	0.834	0.162	0.991	0.042	0.638	0.015	0.122
Chaetedus longiceps Eyles, 1975	Miridae	0.916	0.723	0.717	0.991	0.535	0.542	0.452	0.615
Cletus similis (Blöte, 1935)	Coreidae	0.580	0.873	0.045	0.988	0.014	0.703	0.002	0.074
Coridromius sp.	Miridae	0.828	0.807	0.316	0.992	0.041	0.612	0.017	0.089
Creontiades dilutus (Stäl, 1859)	Miridae	0.662	0.858	0.092	0.990	0.019	0.673	0.004	0.070
Crompus oculatus Stäl, 1874	Lygaeidae	0.396	0.890	0.014	0.981	0.018	0.740	0.003	0.170
Crompus opacus Scudder, 1958	Lygaeidae	0.333	0.884	0.011	0.973	0.018	0.718	0.003	0.107
Cryptorhamphus orbus Stäl, 1860	Cryptorhamphidae	0.406	0.877	0.021	0.979	0.028	0.715	0.005	0.174
Cuspicona sp. 1	Pentatomidae	0.835	0.810	0.338	0.993	0.090	0.592	0.048	0.176
Cuspicona sp. 2	Pentatomidae	0.086	0.827	0.005	0.740	0.092	0.688	0.020	0.298
Cymodema basicornis (Motschulsky, 1863)	Cymidae	0.522	0.838	0.067	0.981	0.030	0.662	0.008	0.097
Dictyotus sp. 1	Pentatomidae	0.378	0.874	0.018	0.974	0.016	0.709	0.003	0.089
Dictyotus sp. 2	Pentatomidae	0.638	0.854	0.059	0.988	0.020	0.672	0.005	0.072
Dieuches maculicollis (Walker, 1872)	Rhyparochromidae	0.353	0.895	0.012	0.985	0.019	0.729	0.003	0.124
Dilompus robustus Scudder, 1957	Artheneidae	0.698	0.873	0.080	0.993	0.020	0.688	0.004	0.098
Dilompus woodwardi Malipatil, 1988	Artheneidae	0.384	0.869	0.020	0.974	0.026	0.697	0.005	0.128
Dindymus versicolor (Herrich-Schaeffer, 1853)	Pyrrhocoridae	0.713	0.843	0.095	0.990	0.032	0.634	0.010	0.087
Diplocysta trilobata Drake & Poor, 1939	Tingidae	0.304	0.894	0.007	0.974	0.018	0.716	0.003	0.102
Eribotes sp.	Pentatomidae	0.620	0.851	0.072	0.985	0.020	0.678	0.005	0.079
Eritingis sp.	Tingidae	0.370	0.875	0.017	0.976	0.017	0.711	0.003	0.091
Euander lacertosus (Erichson, 1842)	Rhyparochromidae	0.660	0.867	0.064	0.991	0.020	0.697	0.004	0.094
Eupolemus sp. 1	Acanthosomatidae	0.490	0.869	0.033	0.984	0.023	0.690	0.005	0.117
Eupolemus sp. 2	Acanthosomatidae	0.456	0.865	0.036	0.979	0.033	0.689	0.008	0.152
Eurynysius sp.	Lygaeidae	0.569	0.887	0.036	0.990	0.016	0.729	0.003	0.123
Eysarcoris distinctus Schouteden, 1906	Pentatomidae	0.652	0.819	0.143	0.984	0.069	0.634	0.024	0.190
Froggattia olivinia Froggatt, 1901	Tingidae	0.321	0.862	0.022	0.962	0.043	0.704	0.009	0.223
Gelonus tasmanicus (Le Guillou, 1841)	Coreidae	0.380	0.873	0.019	0.975	0.016	0.711	0.003	0.089
Halticus minutus Reuter, 1885	Miridae	0.744	0.749	0.366	0.976	0.179	0.573	0.110	0.283
Koscocrompus obscurus Scudder, 1958	Lygaeidae	0.318	0.883	0.011	0.971	0.018	0.715	0.003	0.104
Lethaeini 1	Rhyparochromidae	0.374	0.896	0.012	0.983	0.018	0.733	0.003	0.125
Malandiola sp.	Tingidae	0.321	0.879	0.012	0.969	0.018	0.715	0.003	0.102
Mcateella elongata Hacker, 1927	Piesmatidae	0.781	0.807	0.257	0.989	0.058	0.604	0.026	0.122
Melanacanthus scutellaris (Dallas, 1852)	Alydidae	0.611	0.818	0.120	0.980	0.052	0.627	0.020	0.139
Mictis profana (Fabricius, 1803)	Coreidae	0.563	0.847	0.071	0.981	0.029	0.662	0.008	0.102
Mirini 1	Miridae	0.946	0.807	0.641	0.998	0.092	0.569	0.054	0.150
Mirini 3	Miridae	0.694	0.844	0.105	0.991	0.018	0.670	0.005	0.069
Mirini 4	Miridae	0.533	0.855	0.053	0.984	0.022	0.687	0.005	0.098
Mirini 5	Miridae	0.565	0.845	0.090	0.984	0.061	0.658	0.020	0.203
Mirini 7	Miridae	0.643	0.845	0.114	0.990	0.047	0.649	0.016	0.148
Mirini 8	Miridae	0.523	0.858	0.047	0.984	0.023	0.684	0.005	0.098
Mirini 9	Miridae	0.371	0.871	0.039	0.988	0.084	0.710	0.017	0.347
Mutusca brevicornis (Dallas, 1852)	Alydidae	0.760	0.655	0.540	0.939	0.696	0.548	0.613	0.772
Nezara viridula (Linnaeus, 1758)	Pentatomidae	0.314	0.884	0.012	0.972	0.018	0.722	0.003	0.106
Notius depressus Dallas, 1851	Pentatomidae	0.378	0.886	0.016	0.981	0.018	0.732	0.003	0.139
Nysius caledoniae Distant, 1920	Lygaeidae	0.958	0.772	0.784	0.997	0.306	0.542	0.241	0.382
Nysius vinitor Bergroth, 1891	Lygaeidae	0.896	0.774	0.591	0.994	0.189	0.559	0.128	0.268
Ocirrhoe sp. 1	Pentatomidae	0.139	0.829	0.011	0.856	0.082	0.702	0.018	0.325
Ocirrhoe sp. 2	Pentatomidae	0.351	0.886	0.012	0.978	0.016	0.720	0.003	0.098
Ocirrhoe sp. 3	Pentatomidae	0.755	0.838	0.172	0.993	0.040	0.635	0.014	0.113
Oncocoris geniculatus (Dallas, 1851)	Pentatomidae	0.622	0.861	0.065	0.987	0.021	0.677	0.005	0.085
Ontiscus australis Stäl, 1874	Cymidae	0.342	0.864	0.020	0.969	0.026	0.689	0.005	0.114
Orthotylinae 2	Miridae	0.326	0.886	0.013	0.976	0.017	0.718	0.003	0.103
Orthotylinae 3	Miridae	0.372	0.881	0.015	0.977	0.017	0.715	0.003	0.095
Orthotylinae 7	Miridae	0.404	0.895	0.016	0.984	0.017	0.730	0.003	0.139
Oxycarenus (Oxycarenus) arctatus (Walker, 1872)	Oxycarenidae	0.544	0.880	0.030	0.987	0.014	0.710	0.002	0.081
Oxycarenus (Oxycarenus) luctuosus (Montrouzier, 1861)	Oxycarenidae	0.766	0.874	0.098	0.995	0.019	0.681	0.005	0.086
Pentatomidae 1	Pentatomidae	0.447	0.852	0.049	0.975	0.060	0.674	0.017	0.228
Pentatomidae 20	Pentatomidae	0.443	0.884	0.020	0.982	0.016	0.738	0.003	0.138
Phylinae 1	Miridae	0.822	0.857	0.204	0.996	0.045	0.636	0.017	0.140
Phylinae 7	Miridae	0.401	0.876	0.020	0.977	0.028	0.718	0.005	0.189
Plinthisus (Locutius) woodwardi Slater & Sweet, 1977	Rhyparochromidae	0.628	0.891	0.039	0.992	0.015	0.724	0.002	0.101
Plinthisus sp. 2	Rhyparochromidae	0.532	0.876	0.034	0.987	0.014	0.708	0.002	0.079
Remaudiereana inornata (Walker, 1872)	Rhyparochromidae	0.926	0.753	0.717	0.994	0.357	0.544	0.283	0.440
Sidnia kinbergi (Stäl, 1859)	Miridae	0.918	0.773	0.648	0.996	0.218	0.552	0.155	0.296
Stenophyella macreta Horváth, 1914	Pachygronthidae	0.450	0.646	0.223	0.756	0.443	0.556	0.337	0.552
Stephanitis pyroides (Scott, 1874)	Tingidae	0.165	0.775	0.011	0.739	0.142	0.653	0.044	0.338
Stizocephalus hirsutus Scudder, 1975	Rhyparochromidae	0.337	0.881	0.012	0.975	0.017	0.717	0.003	0.097
Thaumastocoris safordi Noack, Cassis & Rose, 2011	Thaumastocoridae	0.613	0.862	0.058	0.988	0.021	0.682	0.005	0.091
Tingis sp. 1	Tingidae	0.893	0.837	0.356	0.997	0.040	0.613	0.016	0.094
Tingis sp. 2	Tingidae	0.628	0.889	0.038	0.993	0.014	0.718	0.002	0.101
Ulonemia sp.	Tingidae	0.383	0.898	0.013	0.986	0.018	0.729	0.003	0.119
Zanessa rubrovariegata Kirkaldy, 1902	Miridae	0.612	0.865	0.054	0.989	0.014	0.703	0.002	0.068
**Predators**									
Buchananiella whitei Reuter, 1884	Anthocoridae	0.730	0.904	0.052	0.998	0.012	0.701	0.002	0.055
Cermatulus nasalis nasalis (Westwood, 1837)	Pentatomidae	0.826	0.882	0.198	0.998	0.023	0.649	0.007	0.072
Chinoneides tasmaniensis (Gross, 1950)	Berytidae	0.573	0.814	0.145	0.981	0.142	0.614	0.056	0.281
Coranus callosus Stäl, 1874	Reduviidae	0.816	0.878	0.180	0.998	0.017	0.665	0.004	0.060
Deraeocorini 1	Miridae	0.660	0.923	0.026	0.998	0.013	0.705	0.002	0.068
Deraeocorini 2	Miridae	0.615	0.917	0.024	0.997	0.021	0.701	0.004	0.123
Dicrotelus prolixus Erichson, 1842	Reduviidae	0.540	0.895	0.024	0.991	0.030	0.673	0.008	0.116
Dicyphini 1	Miridae	0.643	0.915	0.028	0.997	0.013	0.705	0.002	0.067
Dicyphini 2	Miridae	0.659	0.915	0.028	0.997	0.013	0.704	0.002	0.069
Emesinae 1	Reduviidae	0.780	0.897	0.168	0.999	0.049	0.634	0.018	0.135
Germalus victoriae Bergroth, 1895	Geocoridae	0.648	0.919	0.020	0.997	0.013	0.715	0.002	0.080
Gminatus australis (Erichson, 1842)	Reduviidae	0.800	0.893	0.157	0.999	0.024	0.650	0.007	0.077
Nabis (Tropiconabis) kinbergii Reuter, 1872	Nabidae	0.942	0.882	0.647	1.000	0.286	0.548	0.215	0.370
Oechalia schellenbergii (Guérin, 1831)	Pentatomidae	0.754	0.918	0.048	0.998	0.012	0.708	0.002	0.065
Orius sp.	Anthocoridae	0.781	0.861	0.218	0.997	0.076	0.604	0.037	0.167
Stylogeocoris biroi Montandon, 1913	Geocoridae	0.571	0.920	0.014	0.996	0.014	0.709	0.002	0.075

Table S2. Posterior estimates of the species-specific occurrence and detection probabilities of heteropteran bugs as estimated under the trophic-level model.

Species	Family			99% CI		95% CI		75% CI		50% CI		25% CI		5% CI		1% CI		Effect	
		mean	sd	0.005	0.995	0.025	0.975	0.125	0.875	0.25	0.75	0.375	0.625	0.475	0.525	0.495	0.505	Direction	Strength category
**Herbivores**																			
Amorbus sp.	Coreidae	1.390	0.782	-0.124	4.388	0.213	3.240	0.603	2.234	0.886	1.783	1.078	1.505	1.237	1.315	1.268	1.282	Positive	Very strong
Anischys sp.	Acanthosomatidae	1.313	0.885	-0.859	4.351	-0.236	3.384	0.412	2.224	0.758	1.806	0.995	1.472	1.172	1.271	1.210	1.232	Positive	Strong
Baclozygum depressum Bergroth, 1909	Thaumastocoridae	1.496	0.892	-0.257	4.916	0.127	3.746	0.642	2.465	0.928	1.918	1.130	1.597	1.299	1.390	1.337	1.353	Positive	Very strong
Brentiscerus putoni (Buchanan White, 1878)	Rhyparochromidae	1.332	0.869	-0.816	4.489	-0.169	3.408	0.488	2.277	0.804	1.754	1.029	1.440	1.175	1.257	1.212	1.228	Positive	Strong
Chaetedus longiceps Eyles, 1975	Miridae	1.384	0.734	-0.117	4.176	0.241	3.169	0.674	2.192	0.913	1.735	1.080	1.470	1.229	1.307	1.262	1.277	Positive	Very strong
Cletus similis (Blöte, 1935)	Coreidae	0.887	0.984	-2.113	4.230	-1.124	2.986	-0.098	1.866	0.367	1.402	0.632	1.114	0.832	0.918	0.864	0.884	Positive	Moderate
Coridromius sp.	Miridae	1.162	0.960	-1.400	4.307	-0.613	3.383	0.221	2.171	0.572	1.662	0.866	1.343	1.054	1.149	1.091	1.114	Positive	Strong
Creontiades dilutus (Stäl, 1859)	Miridae	0.841	1.024	-2.143	4.113	-1.159	2.899	-0.270	1.871	0.277	1.396	0.625	1.107	0.823	0.921	0.859	0.882	Positive	Moderate
Crompus oculatus Stäl, 1874	Lygaeidae	0.833	0.934	-1.985	3.900	-1.071	2.758	-0.126	1.777	0.332	1.347	0.583	1.072	0.769	0.871	0.812	0.830	Positive	Moderate
Crompus opacus Scudder, 1958	Lygaeidae	1.296	0.866	-0.593	4.475	-0.103	3.375	0.428	2.214	0.748	1.717	0.988	1.420	1.146	1.231	1.180	1.196	Positive	Strong
Cryptorhamphus orbus Stäl, 1860	Cryptorhamphidae	0.911	0.954	-1.813	4.122	-0.992	2.971	-0.085	1.853	0.359	1.443	0.655	1.139	0.854	0.943	0.892	0.908	Positive	Moderate
Cuspicona sp. 1	Pentatomidae	0.648	0.870	-1.866	3.127	-1.118	2.389	-0.320	1.551	0.113	1.190	0.442	0.922	0.623	0.728	0.661	0.684	Positive	Moderate
Cuspicona sp. 2	Pentatomidae	1.541	0.746	-0.007	4.280	0.349	3.271	0.785	2.330	1.028	1.963	1.230	1.668	1.399	1.484	1.434	1.449	Positive	Very strong
Cymodema basicornis (Motschulsky, 1863)	Cymidae	1.552	0.954	-0.583	5.213	0.060	3.831	0.621	2.556	0.930	2.059	1.166	1.680	1.353	1.454	1.389	1.411	Positive	Very strong
Dictyotus sp. 1	Pentatomidae	1.264	0.936	-0.801	4.810	-0.336	3.498	0.353	2.245	0.695	1.716	0.944	1.394	1.107	1.196	1.142	1.158	Positive	Strong
Dictyotus sp. 2	Pentatomidae	1.140	0.927	-1.388	4.460	-0.549	3.220	0.219	2.128	0.606	1.620	0.853	1.297	1.006	1.100	1.041	1.065	Positive	Strong
Dieuches maculicollis (Walker, 1872)	Rhyparochromidae	0.847	0.925	-1.867	3.755	-1.003	2.837	-0.098	1.775	0.324	1.356	0.591	1.071	0.788	0.885	0.826	0.844	Positive	Moderate
Dilompus robustus Scudder, 1957	Artheneidae	0.475	1.012	-2.726	2.968	-1.779	2.276	-0.683	1.483	-0.091	1.135	0.293	0.859	0.540	0.650	0.585	0.609	Positive	Week
Dilompus woodwardi Malipatil, 1988	Artheneidae	1.299	0.879	-0.686	4.429	-0.158	3.480	0.465	2.235	0.738	1.733	0.957	1.410	1.125	1.211	1.163	1.181	Positive	Strong
Dindymus versicolor (Herrich-Schaeffer, 1853)	Pyrrhocoridae	0.810	1.041	-2.439	4.079	-1.348	3.014	-0.258	1.852	0.274	1.362	0.553	1.066	0.762	0.861	0.800	0.821	Positive	Moderate
Diplocysta trilobata Drake & Poor, 1939	Tingidae	1.319	0.849	-0.673	4.457	-0.103	3.269	0.478	2.203	0.788	1.780	1.000	1.454	1.167	1.250	1.200	1.217	Positive	Strong
Eribotes sp.	Pentatomidae	1.261	0.915	-0.978	4.589	-0.336	3.417	0.368	2.206	0.697	1.734	0.937	1.405	1.101	1.197	1.139	1.160	Positive	Strong
Eritingis sp.	Tingidae	1.295	0.890	-0.815	4.636	-0.205	3.429	0.420	2.251	0.755	1.728	0.979	1.409	1.121	1.212	1.158	1.175	Positive	Strong
Euander lacertosus (Erichson, 1842)	Rhyparochromidae	0.670	0.935	-2.121	3.368	-1.344	2.523	-0.370	1.618	0.135	1.209	0.477	0.949	0.684	0.782	0.726	0.747	Positive	Moderate
Eupolemus sp. 1	Acanthosomatidae	1.210	0.829	-0.704	4.348	-0.150	3.124	0.372	2.093	0.684	1.637	0.907	1.338	1.074	1.156	1.105	1.119	Positive	Strong
Eupolemus sp. 2	Acanthosomatidae	1.109	0.784	-0.780	3.840	-0.275	2.946	0.317	1.918	0.609	1.524	0.832	1.240	0.995	1.075	1.027	1.046	Positive	Strong
Eurynysius sp.	Lygaeidae	0.613	0.954	-2.358	3.413	-1.468	2.479	-0.411	1.577	0.086	1.181	0.402	0.914	0.594	0.712	0.642	0.665	Positive	Moderate
Eysarcoris distinctus Schouteden, 1906	Pentatomidae	1.009	0.781	-0.973	3.794	-0.366	2.823	0.222	1.836	0.528	1.423	0.739	1.154	0.907	0.987	0.936	0.954	Positive	Strong
Froggattia olivinia Froggatt, 1901	Tingidae	0.975	0.919	-1.514	4.204	-0.757	3.009	0.048	1.935	0.438	1.444	0.702	1.130	0.885	0.957	0.917	0.921	Positive	Strong
Gelonus tasmanicus (Le Guillou, 1841)	Coreidae	1.297	0.878	-0.851	4.607	-0.225	3.384	0.454	2.225	0.767	1.722	0.990	1.405	1.152	1.240	1.189	1.205	Positive	Strong
Halticus minutus Reuter, 1885	Miridae	1.106	0.730	-0.396	3.732	-0.048	2.825	0.336	1.892	0.634	1.491	0.841	1.226	0.985	1.067	1.016	1.033	Positive	Strong
Koscocrompus obscurus Scudder, 1958	Lygaeidae	1.279	0.849	-0.580	4.290	-0.106	3.290	0.399	2.204	0.734	1.711	0.971	1.409	1.134	1.221	1.168	1.188	Positive	Strong
Lethaeini 1	Rhyparochromidae	0.708	0.946	-2.113	3.589	-1.266	2.644	-0.267	1.640	0.161	1.239	0.476	0.978	0.686	0.786	0.723	0.746	Positive	Moderate
Malandiola sp.	Tingidae	1.306	0.849	-0.590	4.397	-0.078	3.337	0.467	2.225	0.782	1.720	0.974	1.430	1.143	1.233	1.181	1.200	Positive	Strong
Mcateella elongata Hacker, 1927	Piesmatidae	1.250	0.840	-0.664	4.469	-0.146	3.221	0.419	2.160	0.718	1.654	0.944	1.380	1.109	1.197	1.143	1.162	Positive	Strong
Melanacanthus scutellaris (Dallas, 1852)	Alydidae	1.364	0.952	-0.751	4.882	-0.162	3.672	0.443	2.383	0.775	1.809	1.016	1.468	1.178	1.259	1.206	1.223	Positive	Strong
Mictis profana (Fabricius, 1803)	Coreidae	1.214	0.877	-0.860	4.683	-0.251	3.244	0.360	2.120	0.689	1.622	0.913	1.323	1.076	1.163	1.107	1.126	Positive	Strong
Mirini 1	Miridae	0.746	0.709	-1.243	2.811	-0.652	2.211	-0.028	1.505	0.311	1.187	0.527	0.953	0.697	0.779	0.731	0.747	Positive	Moderate
Mirini 3	Miridae	0.966	1.059	-1.950	4.634	-1.008	3.243	-0.152	2.036	0.382	1.509	0.696	1.191	0.892	0.988	0.931	0.950	Positive	Moderate
Mirini 4	Miridae	1.145	0.946	-1.348	4.417	-0.572	3.325	0.207	2.150	0.597	1.609	0.843	1.295	1.011	1.104	1.053	1.071	Positive	Strong
Mirini 5	Miridae	0.763	0.748	-1.135	3.230	-0.585	2.413	-0.017	1.570	0.277	1.182	0.506	0.933	0.689	0.767	0.721	0.737	Positive	Moderate
Mirini 7	Miridae	0.786	0.793	-1.242	3.405	-0.691	2.517	-0.051	1.619	0.303	1.199	0.557	0.952	0.730	0.794	0.767	0.781	Positive	Moderate
Mirini 8	Miridae	1.082	0.940	-1.394	4.387	-0.705	3.200	0.140	2.065	0.549	1.553	0.812	1.253	0.964	1.052	0.997	1.016	Positive	Strong
Mirini 9	Miridae	0.237	0.906	-2.678	2.826	-1.722	1.948	-0.712	1.175	-0.265	0.792	0.033	0.520	0.216	0.300	0.242	0.263	Positive	Week
Mutusca brevicornis (Dallas, 1852)	Alydidae	1.682	0.662	0.461	4.053	0.680	3.261	0.983	2.420	1.211	2.006	1.406	1.796	1.558	1.636	1.588	1.605	Positive	Extremely strong
Nezara viridula (Linnaeus, 1758)	Pentatomidae	1.284	0.874	-0.815	4.846	-0.201	3.363	0.452	2.169	0.751	1.693	0.976	1.412	1.149	1.239	1.183	1.203	Positive	Strong
Notius depressus Dallas, 1851	Pentatomidae	1.116	0.941	-1.389	4.462	-0.623	3.256	0.198	2.084	0.578	1.595	0.805	1.285	0.992	1.076	1.025	1.042	Positive	Strong
Nysius caledoniae Distant, 1920	Lygaeidae	0.891	0.646	-0.876	2.783	-0.386	2.247	0.180	1.572	0.496	1.271	0.711	1.073	0.857	0.928	0.887	0.900	Positive	Strong
Nysius vinitor Bergroth, 1891	Lygaeidae	0.980	0.614	-0.617	2.936	-0.182	2.309	0.328	1.650	0.602	1.325	0.791	1.117	0.922	0.971	0.946	0.950	Positive	Strong
Ocirrhoe sp. 1	Pentatomidae	1.126	0.721	-0.539	3.753	-0.108	2.889	0.401	1.863	0.679	1.487	0.875	1.250	1.017	1.088	1.043	1.058	Positive	Strong
Ocirrhoe sp. 2	Pentatomidae	1.265	0.853	-0.825	4.202	-0.232	3.279	0.437	2.185	0.717	1.689	0.957	1.409	1.129	1.218	1.163	1.184	Positive	Strong
Ocirrhoe sp. 3	Pentatomidae	1.314	0.897	-0.624	4.737	-0.096	3.480	0.421	2.264	0.751	1.742	0.970	1.414	1.143	1.227	1.174	1.191	Positive	Strong
Oncocoris geniculatus (Dallas, 1851)	Pentatomidae	1.176	0.882	-0.835	4.557	-0.276	3.282	0.297	2.094	0.642	1.617	0.879	1.309	1.041	1.122	1.074	1.091	Positive	Strong
Ontiscus australis Stäl, 1874	Cymidae	1.485	0.833	-0.332	4.602	0.135	3.472	0.666	2.410	0.944	1.908	1.165	1.597	1.312	1.397	1.348	1.363	Positive	Very strong
Orthotylinae 2	Miridae	1.284	0.859	-0.699	4.476	-0.127	3.360	0.467	2.199	0.744	1.710	0.959	1.405	1.123	1.217	1.162	1.182	Positive	Strong
Orthotylinae 3	Miridae	1.285	0.840	-0.850	4.315	-0.154	3.231	0.467	2.188	0.777	1.701	1.000	1.409	1.137	1.224	1.171	1.190	Positive	Strong
Orthotylinae 7	Miridae	0.890	0.965	-1.860	4.026	-0.978	2.944	-0.112	1.886	0.326	1.401	0.640	1.104	0.827	0.906	0.858	0.876	Positive	Moderate
Oxycarenus (Oxycarenus) arctatus (Walker, 1872)	Oxycarenidae	0.777	0.963	-2.243	3.718	-1.229	2.761	-0.244	1.720	0.252	1.330	0.557	1.042	0.762	0.852	0.796	0.815	Positive	Moderate
Oxycarenus (Oxycarenus) luctuosus (Montrouzier, 1861)	Oxycarenidae	0.477	1.023	-3.081	3.092	-1.809	2.278	-0.679	1.476	-0.079	1.091	0.308	0.872	0.559	0.669	0.601	0.624	Positive	Week
Pentatomidae 1	Pentatomidae	0.785	0.785	-1.439	3.254	-0.746	2.436	-0.059	1.581	0.305	1.259	0.557	1.003	0.740	0.824	0.773	0.790	Positive	Moderate
Pentatomidae 20	Pentatomidae	0.900	0.974	-1.863	4.217	-0.952	3.046	-0.141	1.891	0.322	1.408	0.633	1.127	0.828	0.924	0.868	0.887	Positive	Moderate
Phylinae 1	Miridae	0.421	1.034	-2.853	3.113	-1.786	2.301	-0.761	1.467	-0.200	1.083	0.208	0.798	0.471	0.581	0.514	0.533	Positive	Week
Phylinae 7	Miridae	0.928	0.969	-1.803	4.277	-0.924	3.057	-0.052	1.887	0.377	1.415	0.663	1.155	0.862	0.954	0.903	0.919	Positive	Moderate
Plinthisus (Locutius) woodwardi Slater & Sweet, 1977	Rhyparochromidae	0.402	0.964	-2.597	2.878	-1.652	2.099	-0.734	1.374	-0.166	1.031	0.231	0.774	0.468	0.577	0.516	0.533	Positive	Week
Plinthisus sp. 2	Rhyparochromidae	0.961	0.982	-1.789	4.403	-0.927	3.136	-0.009	1.978	0.368	1.459	0.679	1.179	0.887	0.978	0.922	0.940	Positive	Moderate
Remaudiereana inornata (Walker, 1872)	Rhyparochromidae	1.387	0.720	-0.336	3.714	0.134	2.969	0.653	2.199	0.910	1.825	1.106	1.531	1.259	1.345	1.291	1.307	Positive	Very strong
Sidnia kinbergi (Stäl, 1859)	Miridae	1.035	0.686	-0.770	3.434	-0.258	2.533	0.330	1.754	0.612	1.412	0.816	1.191	0.959	1.034	0.987	1.003	Positive	Strong
Stenophyella macreta Horváth, 1914	Pachygronthidae	1.287	0.550	0.233	3.233	0.382	2.600	0.720	1.886	0.919	1.579	1.077	1.379	1.198	1.257	1.223	1.233	Positive	Extremely strong
Stephanitis pyroides (Scott, 1874)	Tingidae	0.010	0.793	-2.431	2.084	-1.692	1.429	-0.881	0.874	-0.473	0.528	-0.189	0.300	0.017	0.109	0.051	0.069	Positive	Very week
Stizocephalus hirsutus Scudder, 1975	Rhyparochromidae	1.278	0.859	-0.664	4.577	-0.150	3.298	0.461	2.183	0.726	1.710	0.950	1.390	1.119	1.208	1.155	1.171	Positive	Strong
Thaumastocoris safordi Noack, Cassis & Rose, 2011	Thaumastocoridae	0.972	0.969	-1.735	3.958	-0.945	3.037	-0.038	2.060	0.403	1.501	0.693	1.202	0.901	0.997	0.941	0.959	Positive	Moderate
Tingis sp. 1	Tingidae	0.716	1.032	-2.491	3.510	-1.565	2.711	-0.422	1.754	0.181	1.320	0.511	1.034	0.724	0.827	0.766	0.788	Positive	Moderate
Tingis sp. 2	Tingidae	0.533	0.956	-2.638	3.073	-1.558	2.316	-0.518	1.463	0.001	1.114	0.358	0.874	0.583	0.684	0.623	0.643	Positive	Moderate
Ulonemia sp.	Tingidae	0.753	0.954	-2.170	3.709	-1.244	2.704	-0.243	1.704	0.230	1.263	0.557	1.001	0.748	0.813	0.766	0.780	Positive	Moderate
Zanessa rubrovariegata Kirkaldy, 1902	Miridae	0.935	0.974	-1.928	3.999	-1.017	3.051	-0.027	1.936	0.367	1.449	0.672	1.144	0.862	0.959	0.902	0.920	Positive	Moderate
**Predators**																			
Buchananiella whitei Reuter, 1884	Anthocoridae	1.112	2.273	-7.416	10.152	-3.482	6.013	-0.525	2.862	0.268	1.932	0.637	1.349	0.912	1.043	0.966	0.988	Positive	Moderate
Cermatulus nasalis nasalis (Westwood, 1837)	Pentatomidae	1.711	1.931	-1.514	10.765	-0.638	6.711	0.232	3.437	0.632	2.204	0.929	1.665	1.189	1.329	1.242	1.271	Positive	Strong
Chinoneides tasmaniensis (Gross, 1950)	Berytidae	1.030	1.128	-0.972	6.083	-0.443	4.180	0.094	1.989	0.371	1.404	0.591	1.039	0.759	0.853	0.801	0.818	Positive	Strong
Coranus callosus Stäl, 1874	Reduviidae	1.102	2.303	-7.256	11.688	-3.350	6.336	-0.584	2.816	0.245	1.776	0.650	1.320	0.910	1.037	0.962	0.990	Positive	Moderate
Deraeocorini 1	Miridae	1.035	2.169	-7.423	10.512	-3.355	5.526	-0.593	2.760	0.213	1.868	0.599	1.308	0.862	0.999	0.914	0.941	Positive	Moderate
Deraeocorini 2	Miridae	1.139	2.185	-5.512	10.513	-2.536	6.597	-0.471	2.830	0.208	1.862	0.587	1.274	0.841	0.976	0.892	0.921	Positive	Moderate
Dicrotelus prolixus Erichson, 1842	Reduviidae	1.994	1.960	-0.997	10.790	-0.225	7.298	0.462	3.827	0.839	2.592	1.145	1.895	1.424	1.562	1.485	1.511	Positive	Strong
Dicyphini 1	Miridae	0.996	2.154	-6.671	9.643	-3.644	5.721	-0.591	2.724	0.193	1.736	0.601	1.276	0.859	0.989	0.909	0.935	Positive	Moderate
Dicyphini 2	Miridae	0.706	1.994	-7.304	7.740	-3.990	4.556	-0.998	2.305	0.021	1.585	0.458	1.135	0.735	0.866	0.787	0.812	Positive	Moderate
Emesinae 1	Reduviidae	1.659	1.473	-1.603	7.588	-0.501	5.377	0.324	3.176	0.722	2.363	1.021	1.789	1.283	1.428	1.338	1.366	Positive	Strong
Germalus victoriae Bergroth, 1895	Geocoridae	1.458	2.115	-4.612	10.934	-1.885	6.656	-0.108	3.247	0.432	2.167	0.789	1.544	1.044	1.190	1.102	1.129	Positive	Moderate
Gminatus australis (Erichson, 1842)	Reduviidae	1.797	1.972	-1.571	11.028	-0.641	7.172	0.267	3.545	0.647	2.446	0.973	1.759	1.233	1.378	1.291	1.318	Positive	Strong
Nabis (Tropiconabis) kinbergii Reuter, 1872	Nabidae	1.180	0.932	-1.504	4.439	-0.461	3.317	0.299	2.134	0.612	1.685	0.856	1.351	1.038	1.138	1.080	1.097	Positive	Strong
Oechalia schellenbergii (Guérin, 1831)	Pentatomidae	0.137	1.813	-7.721	4.124	-4.494	2.865	-1.588	1.731	-0.515	1.146	0.083	0.786	0.402	0.536	0.461	0.490	Positive	Week
Orius sp.	Anthocoridae	1.190	1.726	-3.506	8.130	-1.811	5.416	-0.290	2.792	0.294	1.922	0.629	1.330	0.880	1.010	0.929	0.953	Positive	Moderate
Stylogeocoris biroi Montandon, 1913	Geocoridae	1.779	1.899	-1.387	11.286	-0.432	6.801	0.344	3.415	0.702	2.294	0.992	1.724	1.238	1.384	1.292	1.322	Positive	Strong

Table S3. Posterior estimates for the effect of vegetation volume on herbivorous and predatory heteropteran bug species. The two righmost columns indicate the sign and strength category of each species-specific effect. Grey cells indicate the highest credible interval not containg zero.

Species	Family			99% CI		95% CI		75% CI		50% CI		25% CI		5% CI		1% CI		Effect	
		mean	sd	0.005	0.995	0.025	0.975	0.125	0.875	0.25	0.75	0.375	0.625	0.475	0.525	0.495	0.505	Sign	Strength category
**Herbivores**																			
Amorbus sp.	Coreidae	-1.938	1.356	-6.593	1.077	-4.993	0.307	-3.439	-0.515	-2.692	-1.009	-2.239	-1.417	-1.889	-1.740	-1.831	-1.808	Negative	Strong
Anischys sp.	Acanthosomatidae	-0.264	1.957	-6.605	6.176	-4.333	3.711	-2.180	1.648	-1.326	0.761	-0.749	0.258	-0.329	-0.148	-0.257	-0.222	Negative	Very week
Baclozygum depressum Bergroth, 1909	Thaumastocoridae	-0.094	1.816	-5.580	6.226	-3.749	3.942	-1.866	1.703	-1.076	0.791	-0.550	0.305	-0.225	-0.059	-0.159	-0.124	Negative	Very week
Brentiscerus putoni (Buchanan White, 1878)	Rhyparochromidae	-0.332	1.188	-3.607	3.002	-2.679	2.053	-1.680	0.967	-1.091	0.399	-0.676	0.010	-0.402	-0.267	-0.347	-0.319	Negative	Very week
Chaetedus longiceps Eyles, 1975	Miridae	0.197	0.754	-1.594	2.437	-1.157	1.856	-0.627	1.016	-0.303	0.655	-0.068	0.381	0.109	0.198	0.144	0.161	Positive	Very week
Cletus similis (Blöte, 1935)	Coreidae	-0.876	1.928	-7.124	4.118	-5.230	2.561	-2.944	1.164	-1.907	0.297	-1.243	-0.258	-0.836	-0.655	-0.765	-0.731	Negative	Week
Coridromius sp.	Miridae	-1.547	1.926	-8.476	2.930	-5.948	2.018	-3.528	0.397	-2.568	-0.399	-1.905	-0.931	-1.498	-1.297	-1.418	-1.381	Negative	Moderate
Creontiades dilutus (Stäl, 1859)	Miridae	-1.394	1.995	-8.112	3.736	-5.756	2.198	-3.446	0.596	-2.567	-0.175	-1.814	-0.693	-1.327	-1.110	-1.232	-1.192	Negative	Moderate
Crompus oculatus Stäl, 1874	Lygaeidae	-1.530	1.663	-7.445	2.433	-5.297	1.305	-3.298	0.159	-2.393	-0.474	-1.880	-0.929	-1.460	-1.274	-1.385	-1.348	Negative	Moderate
Crompus opacus Scudder, 1958	Lygaeidae	-0.101	1.935	-5.983	5.835	-3.709	4.082	-2.113	1.890	-1.219	0.978	-0.627	0.395	-0.228	-0.025	-0.139	-0.101	Negative	Very week
Cryptorhamphus orbus Stäl, 1860	Cryptorhamphidae	-0.525	2.018	-8.760	5.364	-4.900	3.272	-2.433	1.358	-1.494	0.606	-0.893	0.050	-0.524	-0.341	-0.453	-0.418	Negative	Very week
Cuspicona sp. 1	Pentatomidae	-0.109	1.749	-4.420	5.627	-3.348	3.785	-1.917	1.751	-1.207	0.927	-0.699	0.259	-0.338	-0.148	-0.261	-0.222	Negative	Very week
Cuspicona sp. 2	Pentatomidae	-0.130	1.388	-4.234	4.704	-2.876	2.762	-1.557	1.222	-0.920	0.654	-0.505	0.206	-0.205	-0.066	-0.146	-0.120	Negative	Very week
Cymodema basicornis (Motschulsky, 1863)	Cymidae	-0.090	1.895	-5.756	6.606	-3.797	3.835	-2.019	1.802	-1.167	0.971	-0.610	0.360	-0.232	-0.047	-0.162	-0.124	Negative	Very week
Dictyotus sp. 1	Pentatomidae	-0.236	2.047	-6.149	7.173	-4.102	3.933	-2.352	1.770	-1.338	0.860	-0.740	0.247	-0.315	-0.132	-0.237	-0.198	Negative	Very week
Dictyotus sp. 2	Pentatomidae	-0.681	2.158	-8.101	5.795	-5.760	3.403	-2.841	1.430	-1.728	0.534	-1.065	-0.034	-0.633	-0.442	-0.554	-0.516	Negative	Week
Dieuches maculicollis (Walker, 1872)	Rhyparochromidae	1.409	1.472	-1.890	6.618	-1.061	4.735	-0.111	3.123	0.422	2.225	0.856	1.660	1.160	1.324	1.225	1.256	Positive	Moderate
Dilompus robustus Scudder, 1957	Artheneidae	1.004	2.081	-4.088	7.690	-2.609	5.622	-1.079	3.394	-0.332	2.131	0.201	1.332	0.627	0.838	0.716	0.755	Positive	Moderate
Dilompus woodwardi Malipatil, 1988	Artheneidae	-1.226	1.752	-7.312	3.037	-5.003	1.929	-3.031	0.578	-2.239	-0.130	-1.616	-0.610	-1.189	-0.994	-1.112	-1.075	Negative	Moderate
Dindymus versicolor (Herrich-Schaeffer, 1853)	Pyrrhocoridae	-2.112	2.062	-9.533	2.578	-6.626	1.254	-4.408	-0.069	-3.201	-0.747	-2.428	-1.299	-1.938	-1.714	-1.844	-1.796	Negative	Strong
Diplocysta trilobata Drake & Poor, 1939	Tingidae	-0.128	1.928	-5.550	5.974	-4.654	3.781	-2.110	1.874	-1.173	0.957	-0.606	0.377	-0.224	-0.037	-0.142	-0.107	Negative	Very week
Eribotes sp.	Pentatomidae	-0.450	2.188	-8.949	5.787	-4.818	3.619	-2.396	1.590	-1.431	0.650	-0.841	0.039	-0.529	-0.336	-0.448	-0.409	Negative	Very week
Eritingis sp.	Tingidae	-0.160	1.916	-5.900	6.199	-3.928	3.892	-2.063	1.826	-1.269	0.894	-0.756	0.297	-0.324	-0.118	-0.238	-0.199	Negative	Very week
Euander lacertosus (Erichson, 1842)	Rhyparochromidae	-1.531	1.897	-7.937	3.241	-6.080	1.892	-3.545	0.344	-2.510	-0.358	-1.850	-0.857	-1.486	-1.272	-1.395	-1.354	Negative	Moderate
Eupolemus sp. 1	Acanthosomatidae	-1.299	1.611	-6.790	2.365	-4.691	1.468	-3.105	0.370	-2.172	-0.259	-1.600	-0.697	-1.207	-1.042	-1.140	-1.108	Negative	Moderate
Eupolemus sp. 2	Acanthosomatidae	-1.199	1.637	-6.745	2.973	-4.977	1.682	-2.943	0.441	-2.101	-0.151	-1.509	-0.608	-1.115	-0.938	-1.041	-1.008	Negative	Moderate
Eurynysius sp.	Lygaeidae	-0.932	1.986	-7.965	4.578	-5.518	2.696	-2.930	0.989	-1.921	0.217	-1.260	-0.323	-0.832	-0.641	-0.742	-0.708	Negative	Week
Eysarcoris distinctus Schouteden, 1906	Pentatomidae	-1.333	1.916	-8.432	3.719	-5.708	2.189	-3.258	0.472	-2.334	-0.214	-1.663	-0.655	-1.207	-1.030	-1.139	-1.102	Negative	Moderate
Froggattia olivinia Froggatt, 1901	Tingidae	-1.612	1.840	-9.194	2.533	-5.979	1.453	-3.528	0.194	-2.572	-0.443	-1.894	-0.936	-1.501	-1.309	-1.427	-1.387	Negative	Moderate
Gelonus tasmanicus (Le Guillou, 1841)	Coreidae	-0.145	1.954	-7.102	5.597	-4.218	3.544	-2.106	1.964	-1.223	0.942	-0.608	0.326	-0.224	-0.043	-0.145	-0.112	Negative	Very week
Halticus minutus Reuter, 1885	Miridae	-1.630	0.993	-4.870	0.833	-3.838	0.044	-2.680	-0.605	-2.230	-0.962	-1.846	-1.248	-1.600	-1.479	-1.554	-1.528	Negative	Strong
Koscocrompus obscurus Scudder, 1958	Lygaeidae	0.026	1.991	-5.686	7.044	-3.770	4.143	-1.962	2.052	-1.097	1.037	-0.533	0.414	-0.163	0.026	-0.083	-0.052	Negative	Extremely week
Lethaeini 1	Rhyparochromidae	1.489	1.482	-1.776	6.840	-0.998	4.970	-0.011	3.012	0.517	2.298	0.922	1.764	1.240	1.406	1.303	1.338	Positive	Moderate
Malandiola sp.	Tingidae	0.117	2.054	-5.605	6.266	-3.772	5.343	-1.915	2.213	-1.039	1.111	-0.503	0.477	-0.111	0.083	-0.042	0.003	na	na
Mcateella elongata Hacker, 1927	Piesmatidae	-2.064	1.682	-7.730	1.885	-5.952	0.850	-3.916	-0.411	-2.936	-0.988	-2.315	-1.432	-1.972	-1.787	-1.891	-1.860	Negative	Strong
Melanacanthus scutellaris (Dallas, 1852)	Alydidae	-0.834	1.789	-7.073	4.238	-4.660	2.641	-2.723	0.931	-1.763	0.178	-1.166	-0.315	-0.816	-0.647	-0.743	-0.710	Negative	Week
Mictis profana (Fabricius, 1803)	Coreidae	-1.283	1.969	-8.950	3.821	-5.564	2.314	-3.450	0.684	-2.293	-0.079	-1.641	-0.623	-1.216	-1.017	-1.140	-1.096	Negative	Moderate
Mirini 1	Miridae	0.368	1.434	-2.674	5.104	-1.997	3.525	-1.122	2.082	-0.608	1.162	-0.191	0.612	0.108	0.263	0.168	0.202	Positive	Very week
Mirini 3	Miridae	-1.040	1.923	-7.773	3.905	-5.329	2.370	-3.048	0.934	-2.070	0.168	-1.433	-0.405	-0.996	-0.797	-0.914	-0.871	Negative	Week
Mirini 4	Miridae	-1.309	1.997	-10.584	3.490	-5.718	2.058	-3.283	0.668	-2.318	-0.087	-1.649	-0.617	-1.207	-1.005	-1.134	-1.087	Negative	Moderate
Mirini 5	Miridae	-2.103	1.798	-9.619	1.600	-6.468	0.647	-4.009	-0.414	-2.936	-0.970	-2.301	-1.370	-1.906	-1.724	-1.829	-1.787	Negative	Strong
Mirini 7	Miridae	1.869	1.759	-1.491	7.993	-0.815	6.318	0.094	3.754	0.673	2.819	1.130	2.094	1.491	1.679	1.566	1.610	Positive	Strong
Mirini 8	Miridae	1.370	1.497	-1.761	7.106	-0.997	5.061	-0.105	2.973	0.399	2.101	0.793	1.566	1.055	1.210	1.120	1.154	Positive	Moderate
Mirini 9	Miridae	1.013	0.845	-1.262	3.810	-0.557	2.866	0.165	1.905	0.470	1.491	0.735	1.211	0.927	1.019	0.964	0.981	Positive	Strong
Mutusca brevicornis (Dallas, 1852)	Alydidae	-0.448	0.471	-1.921	0.689	-1.443	0.418	-0.984	0.068	-0.740	-0.133	-0.566	-0.288	-0.440	-0.387	-0.417	-0.409	Negative	Moderate
Nezara viridula (Linnaeus, 1758)	Pentatomidae	-0.570	1.935	-6.843	5.304	-4.687	3.255	-2.553	1.390	-1.686	0.539	-1.036	-0.029	-0.617	-0.411	-0.527	-0.489	Negative	Week
Notius depressus Dallas, 1851	Pentatomidae	-1.084	1.816	-7.057	3.769	-4.941	2.272	-3.114	0.750	-2.115	0.026	-1.453	-0.474	-1.031	-0.842	-0.961	-0.925	Negative	Week
Nysius caledoniae Distant, 1920	Lygaeidae	1.564	1.461	-1.461	6.360	-0.868	4.812	-0.001	3.363	0.541	2.407	0.974	1.849	1.314	1.480	1.381	1.410	Positive	Moderate
Nysius vinitor Bergroth, 1891	Lygaeidae	-1.596	1.204	-5.408	2.063	-4.281	0.789	-2.850	-0.437	-2.235	-0.880	-1.833	-1.207	-1.594	-1.476	-1.552	-1.523	Negative	Strong
Ocirrhoe sp. 1	Pentatomidae	1.429	1.593	-2.051	7.184	-1.029	5.270	-0.147	3.326	0.360	2.289	0.762	1.634	1.072	1.246	1.137	1.177	Positive	Moderate
Ocirrhoe sp. 2	Pentatomidae	-0.482	1.948	-6.982	5.138	-4.738	3.362	-2.477	1.441	-1.541	0.583	-0.916	0.114	-0.490	-0.296	-0.414	-0.374	Negative	Very week
Ocirrhoe sp. 3	Pentatomidae	-0.120	1.334	-3.708	3.620	-2.691	3.071	-1.518	1.311	-0.920	0.619	-0.520	0.167	-0.255	-0.125	-0.200	-0.176	Negative	Very week
Oncocoris geniculatus (Dallas, 1851)	Pentatomidae	-0.084	2.063	-6.354	6.322	-4.216	4.108	-2.067	2.029	-1.220	0.929	-0.642	0.331	-0.245	-0.053	-0.170	-0.131	Negative	Very week
Ontiscus australis Stäl, 1874	Cymidae	-0.109	1.923	-7.495	5.826	-3.803	3.799	-1.876	1.732	-1.052	0.898	-0.582	0.311	-0.224	-0.063	-0.156	-0.122	Negative	Very week
Orthotylinae 2	Miridae	0.014	2.077	-5.691	9.717	-3.913	4.186	-1.963	1.943	-1.082	1.019	-0.554	0.408	-0.193	0.009	-0.112	-0.078	Negative	Extremely week
Orthotylinae 3	Miridae	-0.098	2.113	-6.024	9.684	-4.152	4.246	-2.087	1.847	-1.189	0.880	-0.683	0.285	-0.311	-0.128	-0.245	-0.208	Negative	Very week
Orthotylinae 7	Miridae	-1.484	1.743	-8.143	2.438	-5.570	1.375	-3.358	0.249	-2.330	-0.376	-1.709	-0.850	-1.384	-1.193	-1.306	-1.270	Negative	Moderate
Oxycarenus (Oxycarenus) arctatus (Walker, 1872)	Oxycarenidae	-1.288	1.850	-7.292	2.914	-5.382	2.193	-3.310	0.625	-2.295	-0.155	-1.646	-0.662	-1.224	-1.048	-1.156	-1.114	Negative	Moderate
Oxycarenus (Oxycarenus) luctuosus (Montrouzier, 1861)	Oxycarenidae	-0.252	1.584	-4.715	4.067	-3.515	2.862	-1.933	1.534	-1.204	0.715	-0.720	0.167	-0.359	-0.203	-0.292	-0.261	Negative	Very week
Pentatomidae 1	Pentatomidae	-1.693	1.977	-8.307	2.844	-6.817	1.464	-3.702	0.130	-2.550	-0.463	-1.875	-0.929	-1.484	-1.276	-1.403	-1.361	Negative	Moderate
Pentatomidae 20	Pentatomidae	-1.381	1.770	-7.194	2.920	-5.228	1.751	-3.453	0.430	-2.363	-0.256	-1.711	-0.737	-1.300	-1.110	-1.222	-1.185	Negative	Moderate
Phylinae 1	Miridae	0.594	1.210	-1.962	5.101	-1.362	3.417	-0.591	1.931	-0.225	1.202	0.109	0.765	0.365	0.502	0.420	0.444	Positive	Week
Phylinae 7	Miridae	-0.485	2.000	-8.105	5.120	-4.671	3.243	-2.463	1.470	-1.482	0.643	-0.884	0.075	-0.500	-0.314	-0.428	-0.390	Negative	Very week
Plinthisus (Locutius) woodwardi Slater & Sweet, 1977	Rhyparochromidae	-0.029	2.205	-5.731	10.088	-4.039	4.443	-2.082	1.971	-1.191	0.954	-0.648	0.392	-0.247	-0.035	-0.153	-0.113	Negative	Very week
Plinthisus sp. 2	Rhyparochromidae	-1.126	1.922	-7.283	3.943	-5.129	2.410	-3.322	0.863	-2.216	0.081	-1.521	-0.463	-1.058	-0.858	-0.979	-0.936	Negative	Week
Remaudiereana inornata (Walker, 1872)	Rhyparochromidae	0.074	0.904	-1.940	3.291	-1.480	2.257	-0.836	1.070	-0.501	0.526	-0.256	0.200	-0.069	0.025	-0.035	-0.015	Negative	Extremely week
Sidnia kinbergi (Stäl, 1859)	Miridae	0.083	1.304	-2.810	4.389	-2.093	3.027	-1.282	1.560	-0.806	0.803	-0.437	0.359	-0.142	0.008	-0.083	-0.052	Negative	Extremely week
Stenophyella macreta Horváth, 1914	Pachygronthidae	-0.801	0.764	-4.110	0.692	-2.635	0.330	-1.562	-0.067	-1.140	-0.311	-0.886	-0.514	-0.754	-0.667	-0.721	-0.701	Negative	Strong
Stephanitis pyroides (Scott, 1874)	Tingidae	2.702	1.543	0.112	9.765	0.650	6.254	1.254	4.434	1.646	3.385	2.017	2.810	2.310	2.463	2.368	2.401	Negative	Extremely strong
Stizocephalus hirsutus Scudder, 1975	Rhyparochromidae	-0.577	1.941	-6.818	5.086	-4.459	3.308	-2.698	1.311	-1.639	0.536	-1.004	-0.033	-0.597	-0.409	-0.524	-0.488	Negative	Week
Thaumastocoris safordi Noack, Cassis & Rose, 2011	Thaumastocoridae	-1.287	1.997	-8.044	3.349	-5.890	2.853	-3.390	0.646	-2.351	-0.104	-1.672	-0.634	-1.232	-1.035	-1.151	-1.114	Negative	Moderate
Tingis sp. 1	Tingidae	0.716	1.266	-1.930	5.162	-1.343	3.607	-0.556	2.153	-0.160	1.433	0.213	0.925	0.467	0.608	0.525	0.557	Positive	Week
Tingis sp. 2	Tingidae	0.286	1.971	-5.222	7.372	-3.258	4.704	-1.616	2.314	-0.924	1.305	-0.356	0.674	0.044	0.247	0.131	0.170	Positive	Very week
Ulonemia sp.	Tingidae	1.460	1.470	-1.723	6.830	-0.988	4.721	-0.058	3.023	0.480	2.273	0.922	1.732	1.223	1.378	1.286	1.318	Positive	Moderate
Zanessa rubrovariegata Kirkaldy, 1902	Miridae	-0.384	1.976	-6.980	5.771	-4.677	3.645	-2.354	1.602	-1.429	0.669	-0.842	0.147	-0.464	-0.261	-0.386	-0.352	Negative	Very week
**Predators**																			
Buchananiella whitei Reuter, 1884	Anthocoridae	-1.260	3.528	-15.277	6.818	-9.313	4.504	-4.653	1.949	-2.839	0.762	-1.661	-0.035	-0.946	-0.613	-0.817	-0.765	Negative	Week
Cermatulus nasalis nasalis (Westwood, 1837)	Pentatomidae	-0.651	3.307	-13.201	8.265	-8.360	5.221	-3.832	2.421	-2.033	1.140	-1.158	0.355	-0.509	-0.228	-0.394	-0.330	Negative	Very week
Chinoneides tasmaniensis (Gross, 1950)	Berytidae	3.169	1.926	-0.192	11.135	0.511	7.876	1.304	5.210	1.881	4.085	2.311	3.364	2.703	2.911	2.785	2.826	Positive	Very strong
Coranus callosus Stäl, 1874	Reduviidae	0.103	3.961	-13.312	14.367	-8.261	8.393	-3.519	3.736	-1.715	1.925	-0.738	0.924	-0.069	0.269	0.062	0.124	Positive	Extremely week
Deraeocorini 1	Miridae	2.404	3.281	-4.737	16.670	-2.261	10.604	-0.486	5.634	0.367	3.903	1.007	2.666	1.599	1.920	1.735	1.792	Positive	Moderate
Deraeocorini 2	Miridae	-1.159	3.259	-13.786	6.500	-9.180	4.162	-4.468	1.891	-2.524	0.751	-1.486	0.003	-0.826	-0.549	-0.722	-0.655	Negative	Very week
Dicrotelus prolixus Erichson, 1842	Reduviidae	0.342	3.530	-11.675	13.152	-6.968	7.749	-2.781	3.512	-1.239	1.837	-0.366	0.930	0.170	0.451	0.292	0.348	Positive	Very week
Dicyphini 1	Miridae	2.334	3.198	-4.186	15.692	-2.253	10.632	-0.513	5.413	0.362	3.615	1.097	2.533	1.627	1.841	1.742	1.792	Positive	Moderate
Dicyphini 2	Miridae	2.492	3.475	-3.946	17.432	-2.140	11.638	-0.408	5.779	0.340	3.746	1.060	2.649	1.616	1.939	1.738	1.794	Positive	Moderate
Emesinae 1	Reduviidae	1.753	2.046	-1.911	10.153	-1.013	7.140	-0.112	3.950	0.392	2.638	0.832	1.852	1.221	1.449	1.319	1.365	Positive	Moderate
Germalus victoriae Bergroth, 1895	Geocoridae	-1.526	3.632	-17.673	6.172	-10.065	4.006	-4.932	1.589	-2.875	0.592	-1.734	-0.154	-1.051	-0.727	-0.917	-0.851	Negative	Week
Gminatus australis (Erichson, 1842)	Reduviidae	-0.876	3.235	-14.231	6.486	-8.890	4.462	-3.996	2.008	-2.120	0.958	-1.175	0.222	-0.570	-0.305	-0.454	-0.406	Negative	Very week
Nabis (Tropiconabis) kinbergii Reuter, 1872	Nabidae	0.561	1.133	-1.768	5.425	-1.072	3.473	-0.423	1.647	-0.122	0.972	0.136	0.630	0.326	0.424	0.363	0.384	Positive	Week
Oechalia schellenbergii (Guérin, 1831)	Pentatomidae	1.123	4.055	-12.068	16.750	-6.491	10.810	-2.306	4.795	-0.841	2.789	0.056	1.702	0.630	0.978	0.761	0.832	Positive	Week
Orius sp.	Anthocoridae	-2.868	3.238	-16.355	3.556	-11.020	1.720	-6.364	-0.048	-4.167	-0.818	-3.005	-1.475	-2.315	-1.994	-2.192	-2.123	Negative	Strong
Stylogeocoris biroi Montandon, 1913	Geocoridae	0.808	3.716	-11.749	14.460	-6.309	8.898	-2.693	4.354	-1.000	2.449	-0.090	1.410	0.519	0.793	0.615	0.675	Positive	Very week

Table S4. Posterior estimates for the effect of plant species diversity on herbivorous and predatory heteropteran bug species. The two righmost columns indicate the sign and strength category of each species-specific effect. Grey cells indicate the highest credible interval not containg zero.

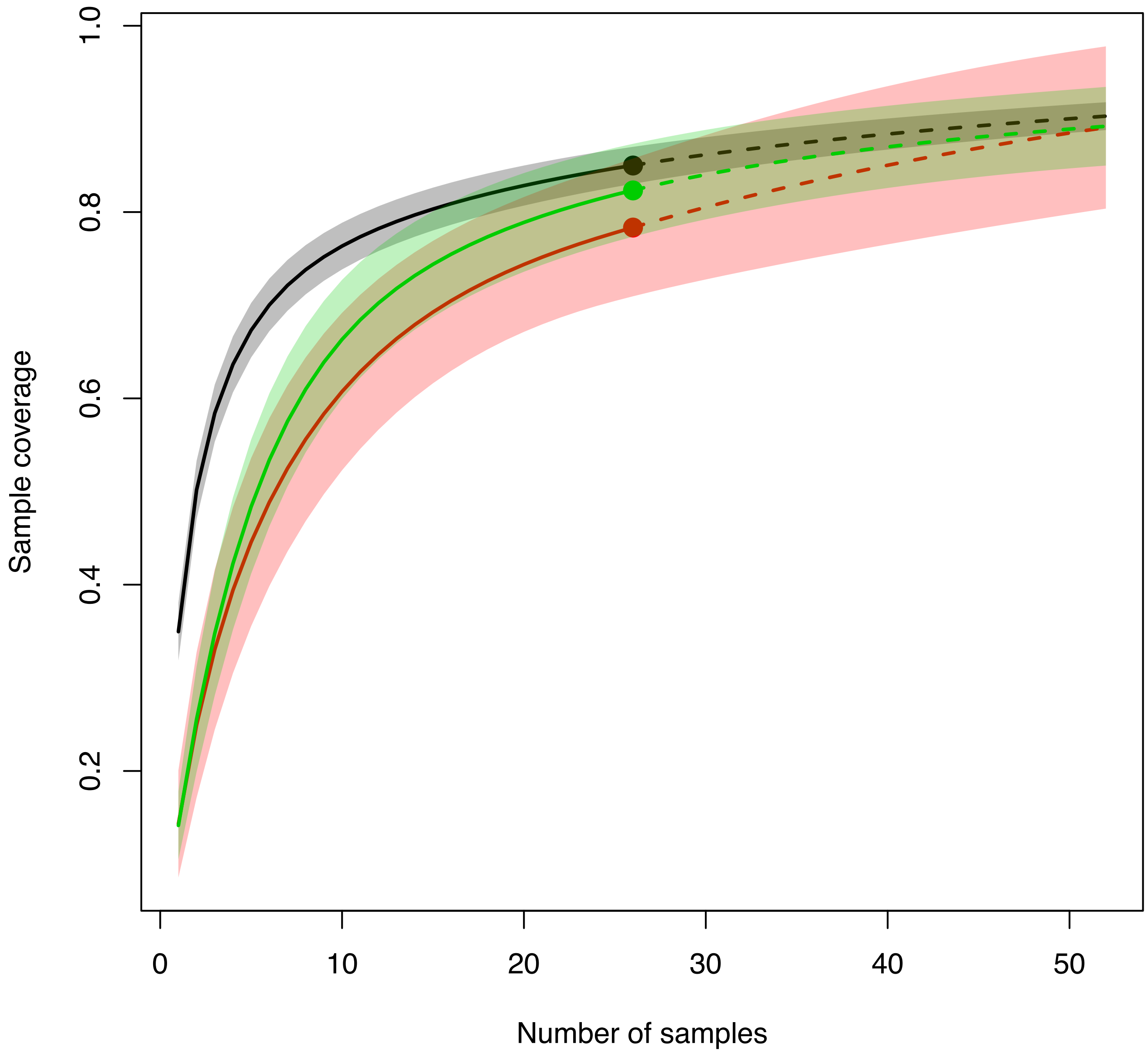

Figure S1. Sample completeness (coverage) curves with 95% confidence intervals for heteropteran bugs sampled within golf courses (black), public parks (red), and residential gardens (green), produced using iNEXT (Hsieh, Ma & Chao 2013), following Chao and Jost (2012). The graph plots sample completeness (as measured by sample coverage) with respect to sample size, where the dot represents our sample size, and the dashed line is an extrapolation of sample coverage up to double the sample size. The graph indicates sufficient sampling effort within each urban green space type, allowing for comparisons across green spaces.

Conserving herbivorous and predatory insects in urban green spaces

Luis Mata, Caragh G. Threlfall, Nicholas S.G. Williams, Amy K. Hahs, Mallik Malipatil, Nigel E. Stork and Stephen J. Livesley.

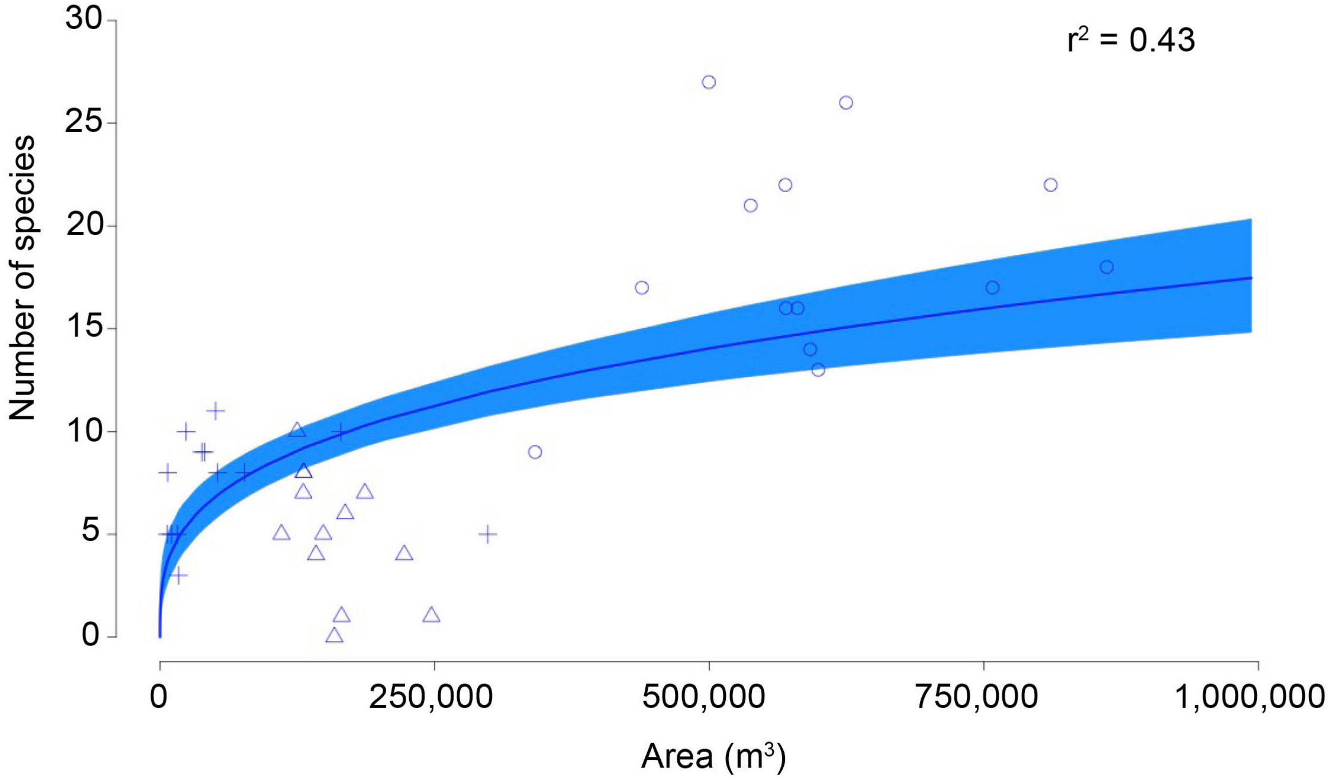

Figure S2. Fit of our data to the power function of the species-area relationship. The mean fit is indicated with a dark blue line, while the shaded light blue region indicates the uncertainty associated with this mean fit (95% credible interval). Symbols represent the empirical data as observed in golf course (circles), park (crosses) and garden (triangles) sites. The value of the coefficient of determination (r2) indicates the proportion of the variance in the estimated number of bug species that is predictable from our model.

Conserving herbivorous and predatory insects in urban green spaces

Luis Mata, Caragh G. Threlfall, Nicholas S.G. Williams, Amy K. Hahs, Mallik Malipatil, Nigel E. Stork and Stephen J. Livesley.

```
#Mata et al. Scientific Reports

#Multi-species site occupancy-detection hierachical model with urban
green space type hyperparameters
#Based on the codes provided by Zipkin et al. (2009) and Mata et al.
(2014)

#Load packages
library(reshape)
library(R2OpenBUGS)

#Read in the data
#setwd(your folder pathway)
sppgc = read.csv("hetgc.csv", header=TRUE, sep=",", na.strings=TRUE)
spppk = read.csv("hetpk.csv", header=TRUE, sep=",", na.strings=TRUE)
spprg = read.csv("hetrg.csv", header=TRUE, sep=",", na.strings=TRUE)
guildsgc = read.csv("guildsgc.csv", header=TRUE, sep=",",
na.strings=c("NA"))
guildspk = read.csv("guildspk.csv", header=TRUE, sep=",",
na.strings=c("NA"))
guildsrg = read.csv("guildsrg.csv", header=TRUE, sep=",",
na.strings=c("NA"))

#Load the species guild data
ggc = guildsgc$guild; gpk = guildspk$guild; grg = guildsrg$guild
agc = which(ggc==1); apk = which(gpk==1); arg = which(grg==1)
hergc = ggc; herpk = gpk; herrg = grg
hergc[-agc] = 0; herpk[-apk] = 0; herrg[-arg] = 0
bgc = which(ggc==2); bpk = which(gpk==2); brg = which(grg==2)
pregc = ggc; prepk = gpk; prerg = grg
pregc[-bgc] = 0; prepk[-bpk] = 0; prerg[-brg] = 0
pregc[bgc] = 1; prepk[bpk] = 1; prerg[brg] = 1

cgc = tapply(sppgc$occ, sppgc$spp2, sum) #How many citings per species
(GC)
cpk = tapply(spppk$occ, spppk$spp2, sum) #How many citings per species
(PK)
crg = tapply(spprg$occ, spprg$spp2, sum) #How many citings per species
(RG)

ugc = as.character(unique(sppgc$spp2)) #Unique species (GC)
upk = as.character(unique(spppk$spp2)) #Unique species (PK)
urg = as.character(unique(spprg$spp2)) #Unique species (RG)

ngc=length(ugc) #Number of observed species (GC)
npk=length(upk) #Number of observed species (PK)
nrg=length(urg) #Number of observed species (RG)

pgc = as.character(unique(sppgc$point)) #Unique sampling locations (GC)
ppk = as.character(unique(spppk$point)) #Unique sampling locations (PK)
prg = as.character(unique(spprg$point)) #Unique sampling locations (RG)

#'zxx' is a 3d array indicating the occurrence of each species per site
and rep
```

```
gcmelt=melt(sppgc, id.var=c("spp2", "point", "rep"), measure.var="occ")
pkmelt=melt(spppk, id.var=c("spp2", "point", "rep"), measure.var="occ")
rgmelt=melt(spprg, id.var=c("spp2", "point", "rep"), measure.var="occ")
zgc=cast(gcmelt, point ~ rep ~ spp2, fill=0)
zpk=cast(pkmelt, point ~ rep ~ spp2, fill=0)
zrg=cast(rgmelt, point ~ rep ~ spp2, fill=0)

#'kxx' is a vector indicating the number of reps at each point j
kgc = as.vector(c(rep(8,13)))
kpk = as.vector(c(rep(2,13)))
krg = as.vector(c(rep(4,13)))

#data for OpenBUGS
bugsdata = list(ngc=ngc, npk=npk, nrg=nrg,
                kgc=kgc, kpk=kpk, krg=krg,
                zgc=zgc, zpk=zpk, zrg=zrg,
                hergc=hergc, herpk=herpk, herrg=herrg,
                pregc=pregc, prepk=prepk, prerg=prerg)

#OpenBUGS code
cat("
   model{

    #Define prior distributions for community-level model parameters
    g.occgc ~ dunif(0,1)
    occgc <- log(g.occgc) - log(1-g.occgc)
    tau.occgc ~ dgamma(0.1,0.1)
    g.occpk ~ dunif(0,1)
    occpk <- log(g.occpk) - log(1-g.occpk)
    tau.occpk ~ dgamma(0.1,0.1)
    g.occrg ~ dunif(0,1)
    occrg <- log(g.occrg) - log(1-g.occrg)
    tau.occrg ~ dgamma(0.1,0.1)
    g.detgc ~ dunif(0,1)
    detgc <- log(g.detgc) - log(1-g.detgc)
    tau.detgc ~ dgamma(0.1,0.1)
    g.detpk ~ dunif(0,1)
    detpk <- log(g.detpk) - log(1-g.detpk)
    tau.detpk ~ dgamma(0.1,0.1)
    g.detrg ~ dunif(0,1)
    detrg <- log(g.detrg) - log(1-g.detrg)
    tau.detrg ~ dgamma(0.1,0.1)

    #Golf courses
    for (i in 1:ngc) {

      #Create priors for species i from the community level prior
distributions
      ogc[i] ~ dnorm(occgc, tau.occgc)
      dgc[i] ~ dnorm(detgc, tau.detgc)

      #Create a loop to estimate the z matrix
      for (j in 1:13) {
        logit(psi.gc[j,i]) <- ogc[i]
```

```
        mu.psi.gc[j,i] <- psi.gc[j,i]
        z.gc[j,i] ~ dbern(mu.psi.gc[j,i])

        #Create a loop to estimate detection for species i at point k
during sampling period k
        for (k in 1:kgc[j]) {
          logit(p.gc[j,k,i]) <-  dgc[i]
          mu.p.gc[j,k,i] <- p.gc[j,k,i]*z.gc[j,i]
          zgc[j,k,i] ~ dbern(mu.p.gc[j,k,i])
        }
      }
    }

    #Parks
    for (i in 1:npk) {

      #Create priors for species i from the community level prior
distributions
      opk[i] ~ dnorm(occpk, tau.occpk)
      dpk[i] ~ dnorm(detpk, tau.detpk)

      #Create a loop to estimate the z matrix
      for (j in 1:13) {
        logit(psi.pk[j,i]) <- opk[i]
        mu.psi.pk[j,i] <- psi.pk[j,i]
        z.pk[j,i] ~ dbern(mu.psi.pk[j,i])

        #Create a loop to estimate detection for species i at point k
during sampling period k
        for (k in 1:kpk[j]) {
          logit(p.pk[j,k,i]) <-  dpk[i]
          mu.p.pk[j,k,i] <- p.pk[j,k,i]*z.pk[j,i]
          zpk[j,k,i] ~ dbern(mu.p.pk[j,k,i])
        }
      }
    }

    #Gardens
    for (i in 1:nrg) {

      #Create priors for species i from the community level prior
distributions
      org[i] ~ dnorm(occrg, tau.occrg)
      drg[i] ~ dnorm(detrg, tau.detrg)

      #Create a loop to estimate the z matrix
      for (j in 1:13) {
        logit(psi.rg[j,i]) <- org[i]
        mu.psi.rg[j,i] <- psi.rg[j,i]
        z.rg[j,i] ~ dbern(mu.psi.rg[j,i])

        #Create a loop to estimate detection for species i at point k
during sampling period k
        for (k in 1:krg[j]) {
```

```
          logit(p.rg[j,k,i]) <-  drg[i]
          mu.p.rg[j,k,i] <- p.rg[j,k,i]*z.rg[j,i]
          zrg[j,k,i] ~ dbern(mu.p.rg[j,k,i])
        }
      }
    }

    #Loop to determine species richness estimates

    for(j in 1:13){
      Nt.gc[j] <- sum(z.gc[j,1:ngc])
      Nh.gc[j] <- inprod(z.gc[j,1:ngc],hergc[1:ngc])
      Np.gc[j] <- inprod(z.gc[j,1:ngc],pregc[1:ngc])
      Nt.pk[j] <- sum(z.pk[j,1:npk])
      Nh.pk[j] <- inprod(z.pk[j,1:npk],herpk[1:npk])
      Np.pk[j] <- inprod(z.pk[j,1:npk],prepk[1:npk])
      Nt.rg[j] <- sum(z.rg[j,1:nrg])
      Nh.rg[j] <- inprod(z.rg[j,1:nrg],herrg[1:nrg])
      Np.rg[j] <- inprod(z.rg[j,1:nrg],prerg[1:nrg])
    }

    #Derived quantities

    Mt.gc <- mean(Nt.gc[])
    Mh.gc <- mean(Nh.gc[])
    Mp.gc <- mean(Np.gc[])
    Mt.pk <- mean(Nt.pk[])
    Mh.pk <- mean(Nh.pk[])
    Mp.pk <- mean(Np.pk[])
    Mt.rg <- mean(Nt.rg[])
    Mh.rg <- mean(Nh.rg[])
    Mp.rg <- mean(Np.rg[])

  }", file="bugsmodel.txt"
)

#Specify the parameters to be monitored (OpenBUGS)
bugspar = list("Mt.gc","Mh.gc","Mp.gc",
               "Mt.pk","Mh.pk","Mp.pk",
               "Mt.rg","Mh.rg","Mp.rg",
               "occgc","occpk","occrg",
               "detgc","detpk","detrg",
               "g.occgc","g.occpk","g.occrg",
               "g.detgc","g.detpk","g.detrg",
               "ogc","opk","org",
               "dgc","dpk","drg")

#Specify the initial values (OpenBUGS)
bugsin = function() {
  psi.gc = runif(1,0.25,1)
  psi.pk = runif(1,0.25,1)
  psi.rg = runif(1,0.25,1)
  list(ogc=rnorm(ngc), opk=rnorm(npk), org=rnorm(nrg),
       dgc=rnorm(ngc), dpk=rnorm(npk), drg=rnorm(nrg),
```

```
       z.gc = matrix(rbinom((ngc)*13, size=1, prob=psi.gc), nrow=13,
ncol=(ngc)),
       z.pk = matrix(rbinom((npk)*13, size=1, prob=psi.pk), nrow=13,
ncol=(npk)),
       z.rg = matrix(rbinom((nrg)*13, size=1, prob=psi.rg), nrow=13,
ncol=(nrg))
      )
}

ni=50000; nb=5000; nc=3; nt=1 #MCMC values for OpenBUGS

#Run OpenBUGS model
modugs = bugs(bugsdata, bugsin, bugspar, "bugsmodel.txt", debug=TRUE,
              n.chains=nc, n.iter=ni, n.burnin=nb, n.thin=nt)

#Figure 1

  #Figure 1a
  jpeg(filename="Fig1a.jpg", width=1100, height=700)
    mu = as.integer(modugs$sims.list$Mh.rg)
    t = table(mu)
    tmax = max(t)
    tmu = as.integer(mean(mu))
    plot(table(0:70), col="white", axes=F, ylab="", ylim=c(0,tmax))
    points(t, col="blue", lwd=3)
    lines(x=c(tmu,tmu), y=c(0,tmax), col="black", lwd=5, type="l")
    box("plot", col="white")
    axis(1, at=c(0,10,20,30,40,50,60,70),
labels=c(0,10,20,30,40,50,60,70),
         cex.axis=3, padj=0.5)
  dev.off()

  #Figure 1b
  jpeg(filename="Fig1b.jpg", width=1100, height=700)
    mu = as.integer(modugs$sims.list$Mp.rg)
    t = table(mu)
    tmax = max(t)
    tmu = as.integer(mean(mu))
    plot(table(0:12), col="white", axes=F, ylab="", ylim=c(0,tmax))
    points(t, col="red", lwd=3)
    lines(x=c(tmu,tmu), y=c(0,tmax), col="black", lwd=5, type="l")
    box("plot", col="white")
    axis(1,at=c(0,1,2,3,4,5,6,7,8,9,10,11,12),
labels=c(0,1,2,3,4,5,6,7,8,9,10,11,12),
         cex.axis=3, padj=0.5)
  dev.off()

  #Figure 1c
  jpeg(filename="Fig1c.jpg", width=1100, height=700)
    mu = as.integer(modugs$sims.list$Mh.pk)
    t = table(mu)
    tmax = max(t)
    tmu = as.integer(mean(mu))
    plot(table(0:70), col="white", axes=F, ylab="", ylim=c(0,tmax))
```

```
    points(t, col="blue", lwd=3)
    lines(x=c(tmu,tmu), y=c(0,tmax), col="black", lwd=5, type="l")
    box("plot", col="white")
    axis(1, at=c(0,10,20,30,40,50,60,70),
labels=c(0,10,20,30,40,50,60,70),
         cex.axis=3, padj=0.5)
  dev.off()

  #Figure 1d
  jpeg(filename="Fig1d.jpg", width=1100, height=700)
    mu = as.integer(modugs$sims.list$Mp.pk)
    t = table(mu)
    tmax = max(t)
    tmu = as.integer(mean(mu))
    plot(table(0:12), col="white", axes=F, ylab="", ylim=c(0,tmax))
    points(t, col="red", lwd=3)
    lines(x=c(tmu,tmu), y=c(0,tmax), col="black", lwd=5, type="l")
    box("plot", col="white")
    axis(1, at=c(0,1,2,3,4,5,6,7,8,9,10,11,12),
labels=c(0,1,2,3,4,5,6,7,8,9,10,11,12),
         cex.axis=3, padj=0.5)
  dev.off()

  #Figure 1e
  jpeg(filename="Fig1a.jpg", width=1100, height=700)
    mu = as.integer(modugs$sims.list$Mh.gc)
    t = table(mu)
    tmax = max(t)
    tmu = as.integer(mean(mu))
    plot(table(0:70), col="white", axes=F, ylab="", ylim=c(0,tmax))
    points(t, col="blue", lwd=3)
    lines(x=c(tmu,tmu), y=c(0,tmax), col="black", lwd=5, type="l")
    box("plot", col="white")
    axis(1, at=c(0,10,20,30,40,50,60,70),
labels=c(0,10,20,30,40,50,60,70),
         cex.axis=3, padj=0.5)
  dev.off()

  #Figure 1f
  jpeg(filename="Fig1f.jpg", width=1100, height=700)
    mu = as.integer(modugs$sims.list$Mp.gc)
    t = table(mu)
    tmax = max(t)
    tmu = as.integer(mean(mu))
    plot(table(0:12), col="white", axes=F, ylab="", ylim=c(0,tmax))
    points(t, col="red", lwd=3)
    lines(x=c(tmu,tmu), y=c(0,tmax), col="black", lwd=5,type="l")
    box("plot", col="white")
    axis(1, at=c(0,1,2,3,4,5,6,7,8,9,10,11,12),
labels=c(0,1,2,3,4,5,6,7,8,9,10,11,12),
         cex.axis=3, padj=0.5)
  dev.off()
```

```
#Mata et al. Scientific Reports

#Multi-species occupancy/detection model with trophic-level
hyperparameters
#Stan model developed in close collaboration with Bob Carpenter (Columbia
University)

#Load packages
library(reshape)
library(rstan)

#Read in the data
#setwd(your folder pathway)
her = read.csv("hether.csv", header=TRUE, sep=",", na.strings=TRUE)
pre = read.csv("hetpre.csv", header=TRUE, sep=",", na.strings=TRUE)
cvd = read.csv("bugcv.csv", header=TRUE, sep=",", na.strings=TRUE)

#Number of citings for each species
cher = tapply(her$occ,her$spp, sum)
cpre = tapply(pre$occ,pre$spp, sum)

#Number of unique species
uher = as.character(unique(her$spp))
upre = as.character(unique(pre$spp))

#Number of observed species
nher = length(uher)
npre = length(upre)

#Number of unique sampling locations
pher = as.character(unique(her$point))
ppre = as.character(unique(pre$point))

#Number of sampled points
jher = length(pher)
jpre = length(ppre)

#zher and zpre are 3d arrays indicating the occurrence of each species
per site and rep

  #Herbivores
  mher = melt(her, id.var=c("spp", "point", "rep"), measure.var="occ")
  zher = cast(mher, point ~ rep ~ spp, fill=0)

  #Predators
  mpre = melt(pre, id.var=c("spp", "point", "rep"), measure.var="occ")
  zpre = cast(mpre, point ~ rep ~ spp, fill=0)

#kher and kpre are vectors of length j(her/pre) indicating the number of
reps at each point j
kher = as.vector(c(rep(8,13), rep(4,13), rep(2,13)))
kpre = as.vector(c(rep(8,13), rep(4,13), rep(2,13)))

#Standardise the covariates
```

```
  #Proportion of plot volume occupied by vegetation
  cv1 = as.vector(cvd$vvol)
  mu1 = mean(cv1)
  sd1 = sd(cv1)
  x1 = as.vector((cv1-mu1)/sd1)

  #Plant species diversity
  cv2 = as.vector(cvd$psd)
  mu2 = mean(cv2)
  sd2 = sd(cv2)
  x2 = as.vector((cv2-mu2)/sd2)

#data for Stan
data.stan = list(nher=nher, kher=kher, zher=zher,
                 npre=npre, kpre=kpre, zpre=zpre,
                 x1=x1, x2=x2, J=39)

#Stan model
cat("

functions {
      int k_eight(int a, int b, int c, int d, int e, int f, int g, int h)
{
        int k8;
        k8 <- a+b+c+d+e+f+g+h;
        return k8;
      }
}

data {
      int<lower=1, upper=39> J;

      int<lower=1, upper=200> nher;
      int<lower=1, upper=200> npre;

      int<lower=2, upper=8> kher[J];
      int<lower=2, upper=8> kpre[J];

      int<lower=0, upper=200> zher[J,8,nher];
      int<lower=0, upper=200> zpre[J,8,npre];

      real<lower=-2, upper=3> x1[J];
      real<lower=-4, upper=4> x2[J];

}

parameters {
      real occ;
      real<lower=0> sigma_occ;
      real det;
      real<lower=0> sigma_det;
      real veg;
      real<lower=0> sigma_veg;
```

```
      real psd;
      real<lower=0> sigma_psd;

      real occher;
      real<lower=0> sigma_occher;
      real occpre;
      real<lower=0> sigma_occpre;

      real dether;
      real<lower=0> sigma_dether;
      real detpre;
      real<lower=0> sigma_detpre;

      real vegher;
      real<lower=0> sigma_vegher;
      real vegpre;
      real<lower=0> sigma_vegpre;

      real psdher;
      real<lower=0> sigma_psdher;
      real psdpre;
      real<lower=0> sigma_psdpre;

      vector[nher] hero;
      vector[npre] preo;

      vector[nher] herd;
      vector[npre] pred;

      vector[nher] herv;
      vector[npre] prev;

      vector[nher] herp;
      vector[npre] prep;

}

model {

      // global hyperpriors
      occ ~ cauchy(0, 2.5);
      sigma_occ ~ cauchy(0, 2.5);
      det ~ cauchy(0, 2.5);
      sigma_det ~ cauchy(0, 2.5);
      veg ~ cauchy(0, 2.5);
      sigma_veg ~ cauchy(0, 2.5);
      psd ~ cauchy(0, 2.5);
      sigma_psd ~ cauchy(0, 2.5);

      // trophic-level hyperpriors
      occher ~ normal(occ, sigma_occ);
      sigma_occher ~ cauchy(0, 2.5);
      occpre ~ normal(occ, sigma_occ);
      sigma_occpre ~ cauchy(0, 2.5);
```

```
      dether ~ normal(det, sigma_det);
      sigma_dether ~ cauchy(0, 2.5);
      detpre ~ normal(det, sigma_det);
      sigma_detpre ~ cauchy(0, 2.5);

      vegher ~ normal(veg, sigma_veg);
      sigma_vegher ~ cauchy(0, 2.5);
      vegpre ~ normal(veg, sigma_veg);
      sigma_vegpre ~ cauchy(0, 2.5);

      psdher ~ normal(psd, sigma_psd);
      sigma_psdher ~ cauchy(0, 2.5);
      psdpre ~ normal(psd, sigma_psd);
      sigma_psd ~ cauchy(0, 2.5);

      // Herbivores

      for (i in 1:nher) {
        hero[i] ~ normal(occher, sigma_occher);
        herd[i] ~ normal(dether, sigma_dether);
        herv[i] ~ normal(vegher, sigma_vegher);
        herp[i] ~ normal(psdher, sigma_psdher);

        for (j in 1:J) {
          if
(k_eight(zher[j,1,i],zher[j,2,i],zher[j,3,i],zher[j,4,i],zher[j,5,i],zher
[j,6,i],zher[j,7,i],zher[j,8,i]) >= 1) {
            1 ~ bernoulli_logit(hero[i] + herv[i] * x1[j] + herp[i] *
x2[j]);

            for (k in 1:kher[j]) {
              zher[j,k,i] ~ bernoulli_logit(herd[i]);
            }
          }

          else {
            real lp1;
            real lp2;
            lp1 <- bernoulli_logit_log(0, hero[i] + herv[i] * x1[j] +
herp[i] * x2[j]);
            lp2 <- bernoulli_logit_log(1, hero[i] + herv[i] * x1[j] +
herp[i] * x2[j]);
            lp2 <- lp2 + kher[j] * bernoulli_logit_log(0, herd[i]);
            increment_log_prob(log_sum_exp(lp1,lp2));
          }
        }
      }

      // Predators

      for (i in 1:npre) {
        preo[i] ~ normal(occpre, sigma_occpre);
        pred[i] ~ normal(detpre, sigma_detpre);
```

```
        prev[i] ~ normal(vegpre, sigma_vegpre);
        prep[i] ~ normal(psdpre, sigma_psdpre);

        for (j in 1:J) {
          if
(k_eight(zpre[j,1,i],zpre[j,2,i],zpre[j,3,i],zpre[j,4,i],zpre[j,5,i],zpre
[j,6,i],zpre[j,7,i],zpre[j,8,i]) >= 1) {
          1 ~ bernoulli_logit(preo[i] + prev[i] * x1[j] + prep[i] *
x2[j]);

          for (k in 1:kpre[j]) {
            zpre[j,k,i] ~ bernoulli_logit(pred[i]);
          }
        }

        else {
          real lp1;
          real lp2;
          lp1 <- bernoulli_logit_log(0, preo[i] + prev[i] * x1[j] +
prep[i] * x2[j]);
          lp2 <- bernoulli_logit_log(1, preo[i] + prev[i] * x1[j] +
prep[i] * x2[j]);
          lp2 <- lp2 + kpre[j] * bernoulli_logit_log(0, pred[i]);
          increment_log_prob(log_sum_exp(lp1,lp2));
        }
      }
    }
}

",file="modelcv.stan")

#Run the Stan model
modcv = stan(file="modelcv.stan", data=data.stan, iter=5000, chains=4)

#Extract posterior estimates from model
fit = extract(modcv)

#Figure 2

  #Figure 2a
  pred = sort(runif(500, 0.1, 0.4))
  cv1 = (pred-mu1)/sd1
  her = mean(fit$occher)
  hercv = mean(fit$vegher)
  pre = mean(fit$occpre)
  precv = mean(fit$vegpre)
  y.her = plogis(her + hercv*cv1)
  y.pre = plogis(pre + precv*cv1)
  jpeg(filename = "Fig2a.jpg", width=1100, height=700)
    par(mai=c(1,2,1,1))
    plot(pred, y.her, type="l", col="blue", ylim=c(0,1),
         cex.axis=1, las=1, ylab="", xlab="", axes=F, lwd=5, lty=1)
    points(pred, y.pre, type="l", lwd=5, col="red", lty=2)
    axis(2, at=c(0,0.2,0.4,0.6,0.8,1), labels=c(0,0.2,0.4,0.6,0.8,"1.0"),
```

```
        las=1, cex.axis=2.5)
    axis(1, at=c(0.1,0.2,0.3,0.4), labels=c(0.1,0.2,0.3,0.4),
         las=1, cex.axis=2.5, padj=0.5)
  dev.off()

  #Figure 2b
  pred = sort(runif(500, 12, 60))
  cv2 = (pred-mu2)/sd2
  her = mean(fit$occher)
  hercv = mean(fit$nather)
  pre = mean(fit$occpre)
  precv = mean(fit$natpre)
  y.her = plogis(her + hercv*cv2)
  y.pre = plogis(pre + precv*cv2)
  jpeg(filename="Fig2b.jpg", width=1100, height=700)
    par(mai=c(1,2,1,1))
    plot(pred, y.her, type="l", col="blue", ylim=c(0,1), cex.axis=1,
         las=1, ylab="", xlab="", axes=F, lwd=5, lty=1)
    points(pred, y.pre, type="l", lwd=5, col="red", lty=2)
    axis(2, at=c(0,0.2,0.4,0.6,0.8,1), labels=c(0,0.2,0.4,0.6,0.8,"1.0"),
         las=1, cex.axis=2.5)
    axis(1, at=c(12,24,36,48,60), labels=c(12,24,36,48,60),
         las=1, cex.axis=2.5, padj=0.5)
  dev.off()

  #Figure 2c
  occ = fit$hero
  a1 = fit$herv
  cv1 = sort(runif(500, 0.1, 0.4))
  cv = (cv1-mu1)/sd1
  l = length(cv1)
  jpeg(filename="Fig2c.jpg", width=1100, height=700)
    par(mfrow=c(1,1), mai=c(1,2,1,1))
    plot(cv1, cv1, type="l", ylim=c(0,1), xlim=c(0.1,0.4), main="",
xlab="", ylab="",
         col="white",las=1,cex.axis=1.9,axes=F)
    z = c(45,68,2,4,7,10,15,16,19,21,22,23,25,26,28,29,30,31,32,
          34,35,36,37,40,43,46,47,48,49,50,51,52,53,55,56,67,70,
          1,3,5,13,14,54,66) #Herbivores showing a strong response to
vvol
    for (i in 1:length(z)) {
      q = z[i]
      W = mean(occ[,q]) + mean(a1[,q])*cv
      lines(cv1, plogis(W), type="l", ylim=c(0,1), xlim=c(0.1,0.4),
col="blue", lwd=2)
    }
    axis(2, at=c(0,0.2,0.4,0.6,0.8,1), labels=c(0,0.2,0.4,0.6,0.8,"1.0"),
         las=1, cex.axis=2.5, padj=.5)
    axis(1, at=c(0.1,0.2,0.3,0.4), labels=c(0.1,0.2,0.3,0.4),
         las=1, cex.axis=2.5, padj=.5)
  dev.off()

  #Figure 2d
  occ = fit$hero
```

```
  a1 = fit$hern
  cv2 = sort(runif(500, 12, 60))
  cv = (cv2-mu2)/sd2
  l = length(cv2)
  jpeg(filename="Fig2d", width=1100, height=700)
    par(mfrow=c(1,1), mai=c(1,2,1,1))
    plot(cv2, cv2, type="l", ylim=c(0,1), xlim=c(12,60), main="",
xlab="",
         ylab="", col="white", las=1, cex.axis=1.9, axes=F)
    z = c(69,1,20,31,35,41,42,44,49,68) ##Herbivores showing a strong
response to psd
    for (i in 1:length(z)) {
      q = z[i]
      W = mean(occ[,q]) + mean(a1[,q])*cv
      lines(cv2, plogis(W), type="l", ylim=c(0,1), xlim=c(12,60),
col="blue", lwd=2)
    }
    axis(2, at=c(0,0.2,0.4,0.6,0.8,1), labels=c(0,0.2,0.4,0.6,0.8,"1.0"),
         las=1, cex.axis=2.5, padj=.5)
    axis(1, at=c(12,24,36,48,60), labels=c(12,24,36,48,60),
         las=1, cex.axis=2.5, padj=.5)
  dev.off()

  #Figure 2e
  occ = fit$preo
  a1 = fit$prev
  cv1 = sort(runif(500, 0.1, 0.4))
  cv = (cv1-mu1)/sd1
  l = length(cv1)
  jpeg(filename="Fig2e", width=1100, height=700)
    par(mfrow=c(1,1), mai=c(1,2,1,1))
    plot(cv1, cv1, type="l", ylim=c(0,1), xlim=c(0.1,0.4), main="",
xlab="",
         ylab="", col="white", las=1, cex.axis=1.9, axes=F)
    z = c(2,3,7,10,12,13,16) #Predators showing a strong response to vvol
    for (i in 1:length(z)) {
      q = z[i]
      W = mean(occ[,q]) + mean(a1[,q])*cv
      lines(cv1, plogis(W), type="l", ylim=c(0,1), xlim=c(0.1,0.4),
col="red", lwd=2)
    }
    axis(2, at=c(0,0.2,0.4,0.6,0.8,1), labels=c(0,0.2,0.4,0.6,0.8,"1.0"),
         las=1, cex.axis=2.5, padj=.5)
    axis(1, at=c(0.1,0.2,0.3,0.4), labels=c(10,20,30,40),
         las=1, cex.axis=2.5, padj=.5)
  dev.off()

  #Figure 2f
  occ = fit$preo
  a1 = fit$pren
  cv2 = sort(runif(500, 12, 60))
  cv = (cv2-mu2)/sd2
  l = length(cv2)
  jpeg(filename="Fig2f", width=1100, height=700)
```

```
    par(mfrow=c(1,1), mai=c(1,2,1,1))
    plot(cv2, cv2, type="l", ylim=c(0,1), xlim=c(12,60), main="",
xlab="",
         ylab="", col="white", las=1, cex.axis=1.9, axes=F)
    z = c(3,15) #Predators showing a strong response to psd
    for (i in 1:length(z)) {
      q = z[i]
      W = mean(occ[,q]) + mean(a1[,q])*cv
      lines(cv2, plogis(W), type="l", ylim=c(0,1), xlim=c(12,60),
col="red", lwd=2)
    }
    axis(2, at=c(0,0.2,0.4,0.6,0.8,1), labels=c(0,0.2,0.4,0.6,0.8,"1.0"),
         las=1, cex.axis=2.5, padj=.5)
    axis(1, at=c(12,24,36,48,60), labels=c(12,24,36,48,60),
         las=1, cex.axis=2.5, padj=.5)
  dev.off()

#Figure 3

  #Figure 3a
  predv = sort(runif(500, 0.1, 0.4))
  predp = sort(runif(500, 12, 60))
  cv1 = (predv-mu1)/sd1
  cv2 = (predp-mu2)/sd2
  her = mean(fit$occher)
  herv = mean(fit$vegher)
  herp = mean(fit$nather)
  yher = matrix(NA, nrow=x, ncol=y)
  for (i in 1:x){
    for (j in 1:y){
      yher[i,j] = plogis(her + herv*cv1[i] + herp*cv2[j])
    }
  }
  grid = data.frame(x1 = seq(from=min(predv), to=max(predv),
length.out=x),
                    x2 = seq(from=min(predp), to=max(predp),
length.out=y))
  jpeg(filename="Fig3a", width=1100, height=700)
    par(mai=c(1,2,1,1))
    image(grid$x1, grid$x2, yher,axes=F, col=rev(heat.colors(100)),
          ylab="", xlab="", xlim=c(0.1,0.4), ylim=c(12,60))
    contour(grid$x1, grid$x2, yher, axes=F,
levels=c(0.1,0.2,0.3,0.4,0.6,0.8,0.9,0.95,0.98),

labels=c("0.10","0.20","0.30","0.40","0.60","0.80","0.90",0.95,0.98),
            drawlabels=T, labcex=1.5, lwd=2, method="edge", add=T)
    axis(1, at=c(0.1,0.2,0.3,0.4), labels=c(10,20,30,40),
         las=1, cex.axis=2.5, padj=0.5, line=0.5)
    axis(2, at=c(12,24,36,48,60), labels=c(12,24,36,48,60),
         las=1, cex.axis=2.5, padj=0.5, line=0.5)
    gc.veg = cvd$v.tot[1:13]
    pk.veg = cvd$v.tot[27:39]
    rg.veg = cvd$v.tot[14:26]
    gc.psd = cvd$pd[1:13]
```

```
    pk.psd = cvd$pd[27:39]
    rg.psd = cvd$pd[14:26]
    rect(xleft=min(gc.veg), ybottom=min(gc.psd), xright=max(gc.veg),
         ytop=max(gc.psd), lwd=3)
    rect(xleft=min(pk.veg), ybottom=12.2, xright=max(pk.veg),
         ytop=max(pk.psd), lty="dotted", lwd=3)
    rect(xleft=0.101, ybottom=min(rg.psd), xright=max(rg.veg),
         ytop=59.8, lty="dashed", lwd=3)
  dev.off()

  #Figure 3b
  predv = sort(runif(500, 0.1, 0.4))
  predp = sort(runif(500, 12, 60))
  cv1 = (predv-mu1)/sd1
  cv2 = (predp-mu2)/sd2
  pre = mean(fit$occpre)
  prev = mean(fit$vegpre)
  prep = mean(fit$natpre)
  ypre = matrix(NA, nrow=x, ncol=y)
  for (i in 1:x){
    for (j in 1:y){
      yher[i,j] = plogis(pre + prev*cv1[i] + prep*cv2[j])
    }
  }
  grid = data.frame(x1 = seq(from=min(predv), to=max(predv),
length.out=x),
                    x2 = seq(from=min(predp), to=max(predp),
length.out=y))
  jpeg(filename="Fig3b", width=1100, height=700)
    par(mai=c(1,2,1,1))
    image(grid$x1, grid$x2, yher,axes=F, col=rev(heat.colors(100)),
          ylab="", xlab="", xlim=c(0.1,0.4), ylim=c(12,60))
    contour(grid$x1, grid$x2, yher, axes=F,
levels=c(0.1,0.2,0.3,0.4,0.6,0.8,0.9,0.95,0.98),

labels=c("0.10","0.20","0.30","0.40","0.60","0.80","0.90",0.95,0.98),
            drawlabels=T, labcex=1.5, lwd=2, method="edge", add=T)
    axis(1, at=c(0.1,0.2,0.3,0.4), labels=c(10,20,30,40),
         las=1, cex.axis=2.5, padj=0.5, line=0.5)
    axis(2, at=c(12,24,36,48,60), labels=c(12,24,36,48,60),
         las=1, cex.axis=2.5, padj=0.5, line=0.5)
    gc.veg = cvd$v.tot[1:13]
    pk.veg = cvd$v.tot[27:39]
    rg.veg = cvd$v.tot[14:26]
    gc.psd = cvd$pd[1:13]
    pk.psd = cvd$pd[27:39]
    rg.psd = cvd$pd[14:26]
    rect(xleft=min(gc.veg), ybottom=min(gc.psd), xright=max(gc.veg),
         ytop=max(gc.psd), lwd=3)
    rect(xleft=min(pk.veg), ybottom=12.2, xright=max(pk.veg),
         ytop=max(pk.psd), lty="dotted", lwd=3)
    rect(xleft=0.101, ybottom=min(rg.psd), xright=max(rg.veg),
         ytop=59.8, lty="dashed", lwd=3)
  dev.off()
```

```
#Species area relationship model

#Load R packages
library(reshape)
library(jagsUI)

#Read in the data
#setwd(your folder pathway)
sppgc = read.csv("hetgc.csv", header=TRUE,sep=",",na.strings=TRUE)
spppk = read.csv("hetpk.csv", header=TRUE,sep=",",na.strings=TRUE)
spprg = read.csv("hetrg.csv", header=TRUE,sep=",",na.strings=TRUE)
cvd = read.csv("bugcv.csv", header=TRUE, sep=",", na.strings=TRUE)

cgc = tapply(sppgc$occ, sppgc$spp2, sum) #How many citings per species
(GC)
cpk = tapply(spppk$occ, spppk$spp2, sum) #How many citings per species
(PK)
crg = tapply(spprg$occ, spprg$spp2, sum) #How many citings per species
(RG)

ugc = as.character(unique(sppgc$spp2)) #Unique species (GC)
upk = as.character(unique(spppk$spp2)) #Unique species (PK)
urg = as.character(unique(spprg$spp2)) #Unique species (RG)

ngc=length(ugc) #Number of observed species (GC)
npk=length(upk) #Number of observed species (PK)
nrg=length(urg) #Number of observed species (RG)

pgc = as.character(unique(sppgc$point)) #Unique sampling locations (GC)
ppk = as.character(unique(spppk$point)) #Unique sampling locations (PK)
prg = as.character(unique(spprg$point)) #Unique sampling locations (RG)

#'zxx' is a 3d array indicating the occurrence of each species per site
and rep
gcmelt=melt(sppgc,id.var=c("spp2", "point", "rep"), measure.var="occ")
pkmelt=melt(spppk,id.var=c("spp2", "point", "rep"), measure.var="occ")
rgmelt=melt(spprg,id.var=c("spp2", "point", "rep"), measure.var="occ")
zgc=cast(gcmelt, point ~ rep ~ spp2, fill=0)
zpk=cast(pkmelt, point ~ rep ~ spp2, fill=0)
zrg=cast(rgmelt, point ~ rep ~ spp2, fill=0)

#Add up the number of species per site that were oberved one or more
times
gc = matrix(NA, nrow=13, ncol=ngc)
for (i in 1:13) {
  for (j in 1:ngc) {
    gc[i,j] = sum(zgc[i,1:8,j])
  }
}
gc.sr = rowSums(gc >=1)

pk = matrix(NA, nrow=13, ncol=npk)
for (i in 1:13) {
  for (j in 1:npk) {
```

```
    pk[i,j] = sum(zpk[i,1:2,j])
  }
}
pk.sr = rowSums(pk >=1)

rg = matrix(NA, nrow=13, ncol=nrg)
for (i in 1:13) {
  for (j in 1:nrg) {
    rg[i,j] = sum(zrg[i,1:4,j])
  }
}
rg.sr = rowSums(rg >=1)

S = c(gc.sr, pk.sr, rg.sr)

#Define site area as a variable
A = as.vector(cvd$area)

#Define AP as a vector to draw predictions across the full range of the
area gradient
AP = as.integer(c(runif(20, 0, 10), runif(20, 10, 100), runif(20, 100,
1000), runif(20, 1000, 10000),
                  runif(20, 10000, 100000), runif(20, 100000, 1000000)))

#data for the JAGS
jdata = list(S=S, A=A, AP=AP)
str(jdata)

#JAGS code
cat("
    model{

      #Priors
      c ~ dunif(0,1)
      z ~ dunif(0,1)

      #Likelihood
      for (i in 1:39) {
        S[i] ~ dpois(lambda[i])
        lambda[i] <- c*(A[i]^z)
      }

      #Derived quantities
      for (i in 1:120) {
        SP[i] <- c*(AP[i]^z)
      }

    }", file="sarmodel.txt"
)

#Initial values
inits = function() list(c = runif(1,0,1), z = runif(1,0,1))

#Parameters monitored
```

```
params <- c("c", "z", "lambda", "SP")

#MCMC settings
ni = 50000
nt = 1
nb = 5000
nc = 3

#Call JAGS from R
mod <- jags(jdata, inits, params, "sarmodel.txt",
            n.chains = nc, n.thin = nt, n.iter = ni, n.burnin = nb)
names(mod)
print(mod, 2)

#Assign model's posteriors to variables

c = mod$mean$c
cl = mod$q2.5$c
ch = mod$q97.5$c

z = mod$mean$z
zl = mod$q2.5$z
zh = mod$q97.5$z

sm = mod$mean$SP
sl = mod$q2.5$SP
sh = mod$q97.5$SP

#Calculate species richness with model's estimates
SE = as.integer(c*(A^z))

#Correlate estimations with observations (r2)
cor = summary(lm(SE~S))
cor

#Figure S2
#Plot fit of data with power function of SAR including uncertainty
derived from model

jpeg(filename="Fig. S2", width=1100, height=700)
  plot(sort(AP), sort(sm), type="l", ylim=c(0,30), xlim=c(0,1000000),
main="", xlab="", ylab="",
       col="white", las=1, cex.axis=1.9, axes=F, lwd=3)
  polygon(c(rev(sort(AP)),sort(AP)), c(rev(sort(sl)),sort(sh)),
density=NA, col="dodgerblue")
  points(sort(AP), sort(sm), type="l", ylim=c(0,30), xlim=c(0,1000000),
main="", xlab="", ylab="",
         col="blue", las=1, cex.axis=1.9, lwd=3)
  points(A[1:13], S[1:13], col="blue", pch=1, cex=2)
  points(A[14:26], S[14:26], col="blue", pch=2, cex=2)
  points(A[27:39], S[27:39], col="blue", pch=3, cex=2)
  axis(2, at=c(0,5,10,15,20,25,30), labels=c(0,5,10,15,20,25,30), las=1,
cex.axis=2.5, padj=.5)
```

```
  axis(1, at=c(0,250000,500000,750000,1000000),
labels=c(0,250000,500000,750000,1000000), las=1, cex.axis=2.5, padj=.5)
dev.off()
```
